# Supplementary material for: First-in-class transactivator-free, doxycycline-inducible IL-18-engineered CAR-T cells for relapsed/refractory B cell lymphomas
Source: Mol Ther Nucleic Acids. 2024 Aug 15;35(4):102308. doi: 10.1016/j.omtn.2024.102308 (PMC11617245; doi:10.1016/j.omtn.2024.102308)
Supplement: Document S1. Figures S1–S7 [file mmc1.pdf]

OMTN, Volume *35*

Supplemental information

First-in-class transactivator-free, doxycycline-inducible IL-18-engineered CAR-T cells for relapsed/refractory B cell lymphomas

Pedro Justicia-Lirio, María Tristán-Manzano, Noelia Maldonado-Pérez, Carmen Barbero-Jiménez, Marina Cortijo-Gutiérrez, Kristina Pavlovic, Francisco J. Molina-Estevez, Pilar Muñoz, Ana Hinckley-Boned, Juan R. Rodriguez-Madoz, Felipe Prosper, Carmen Griñán-Lison, Saúl A. Navarro-Marchal, Carla Panisello, Julia Muñoz-Ballester, Pedro A. González-Sierra, Concha Herrera, Juan A. Marchal, and Francisco Martín

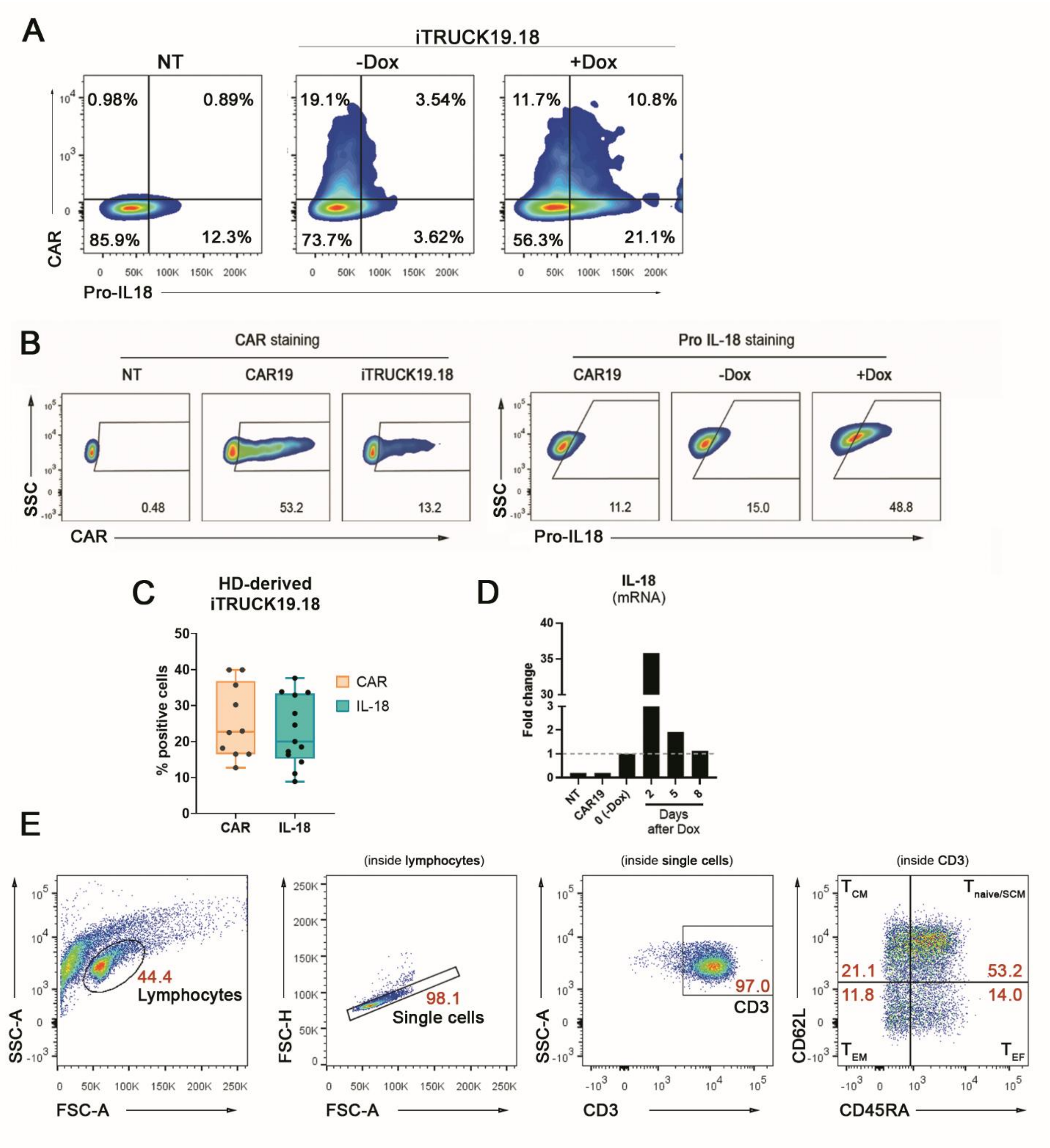

Fig. S1. Transduction efficiencies, inducibility and phenotype analysis of iTRUCK19.18 cells. (A) Representative dot-plots of co-staining of NT, iTRUCKs (-Dox) and (+Dox) 10 days after transduction. Detection was evaluated with anti-pro-IL-18 antibody (as described in M&M) and CAR-19 reagent from Miltenyi Biotech following manufacturer's instructions**.** (B) Representative dot-plots of CAR19 and iTRUCK19.18 cells showing expression of CAR (left) and pro-IL-18

(right). (C) Graph showing the percentage of CAR+ (orange bar) and IL-18 (green bar) of 10 different productions of iTRUCK19.18 cells from healthy donor, measured in the presence of Dox at day 10 post-transduction. (D) Graph showing fold change of IL-18 RNA levels in untransduced T cells, CAR19 cells and iTRUCK19.18 cells at different times upon Dox addition (2 days) and removal (days 5 and 8; days 3 and 6 without Dox). (E) Gating strategy to analyze the phenotype and subpopulations of T cells. After selecting the lymphocytes gate regarding to FSC and SSC, doubles exclusion and confirming CD3, four populations were established according to the expression of CD45RA and CD62L. $T_{naive/SCM}$: CD45RA+CD62L+; T_{CM}: CD45RA-CD62L-; T_{EM}: CD45RA-CD62L-; T_{EF}: CD45RA+CD62L-.

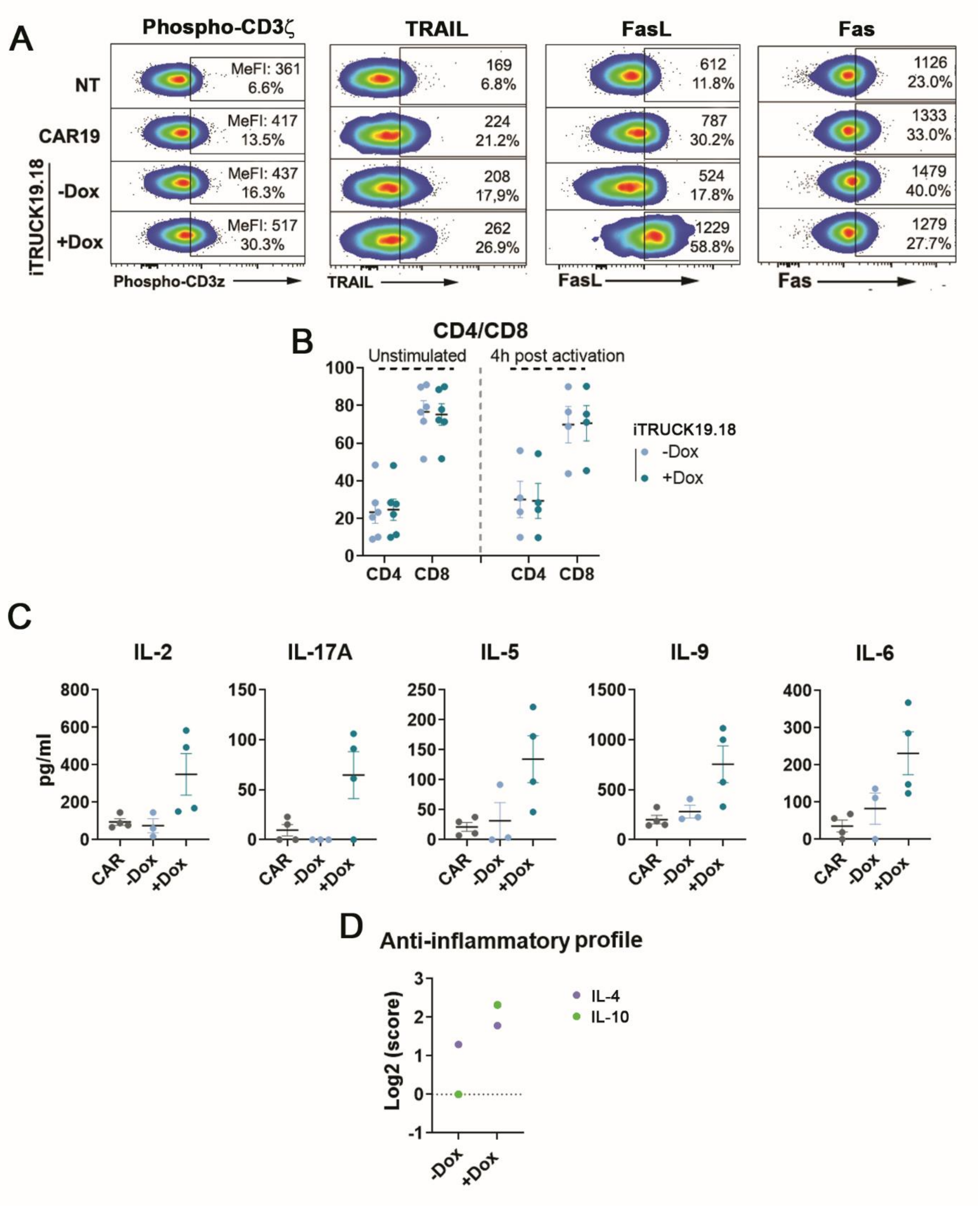

Fig. S2 Characterization of AICD markers and cytokines secretion of iTRUCK19.18 cells. (A) Representative dot-plots of phosphorylated-CD3ζ, TRAIL, FasL and Fas of untranduced T cells (NT; top Plots), CAR19 cells (second-top), iTRUCK19.18 cells -Dox (second-bottom Plots) and iTRUCK19.18 cells +Dox (+50 ng/ml Dox) (bottom plots) after manufacturing (basal

conditions). (B) Percentage of CD4/CD8 inside the iTRUCK19.18 population in the absence (-Dox) or presence of 50ng/ml of Dox (+Dox) at basal state (left; n=6) and 4h post stimulation via CD3/CD28 (right; n=4). (C) Quantification of proinflammatory cytokine secretion by iTRUCK19.18 cells (from left to right: IL-2, IL-17A, IL-5, IL-9, and IL-6) at basal state (n=4). (D) Anti-inflammatory index of iTRUCK19.18 cells (IL-4 and IL-10) at basal level (n=2) in the absence (left) and presence (right) of 50 ng/ml of Dox at basal level.

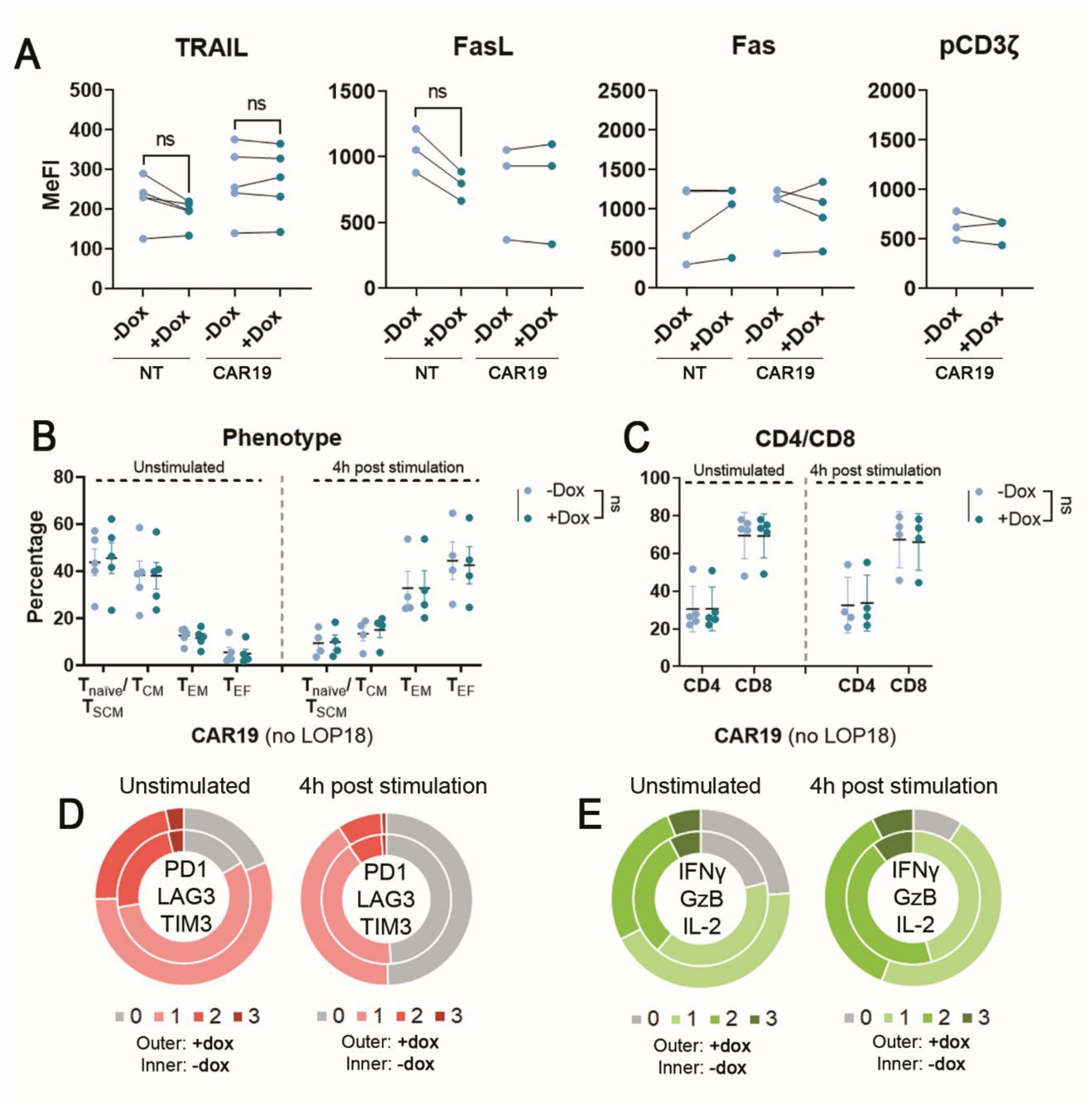

Fig. S3 Doxycycline does not alter the physiology of CAR19 cells. (A) Expression of AICD markers and phosphorylated-CD3ζ (from left to right) in NT and CAR19 cells at basal state in the presence (50 ng/ml) or absence of Dox at basal conditions after manufacturing. TRAIL (n=5), FasL (n=3), Fas (n=4) and pCD3ζ (n=3) (two-tailed paired t test). (B) Percentage of positive cells of $T_{Naïve/SCM}$, T_{CM}, T_{EM} and T_{EF} at basal state (n=5) (left) and 4h post stimulation (n=4) (right) of CAR19 cells with (50 ng/ml) and without Dox. (C) Percentage of CD4 and CD8 of CAR19 cells with (50 ng/ml) and without Dox at basal state (n=5) (left) and 4h post stimulation (n=4) (right). (D) Pie charts showing the proportion of CAR19 cells with (50 ng/ml) (outer circles) and without

(inner circles) Dox expressing 0, 1, 2, or 3 inhibitory receptors (PD1, LAG3, and TIM3) at basal state (n=4) (left) and 4h after stimulation (n=4) (right). (E) Pie charts showing the proportion of CAR19 cells with (50 ng/ml) (outer circles) and without (inner circles) Dox expressing 0, 1, 2, or 3 activation markers (IFNγ, Granzyme B, and IL-2) at basal state (n=4) (left) and 4h post stimulation (n=4) (right). Activation was performed using T cell TransAct (Miltenyi) (via CD3/CD28). ns: non-significant (two-tailed paired t test for A, one-tailed paired t test for B and C).

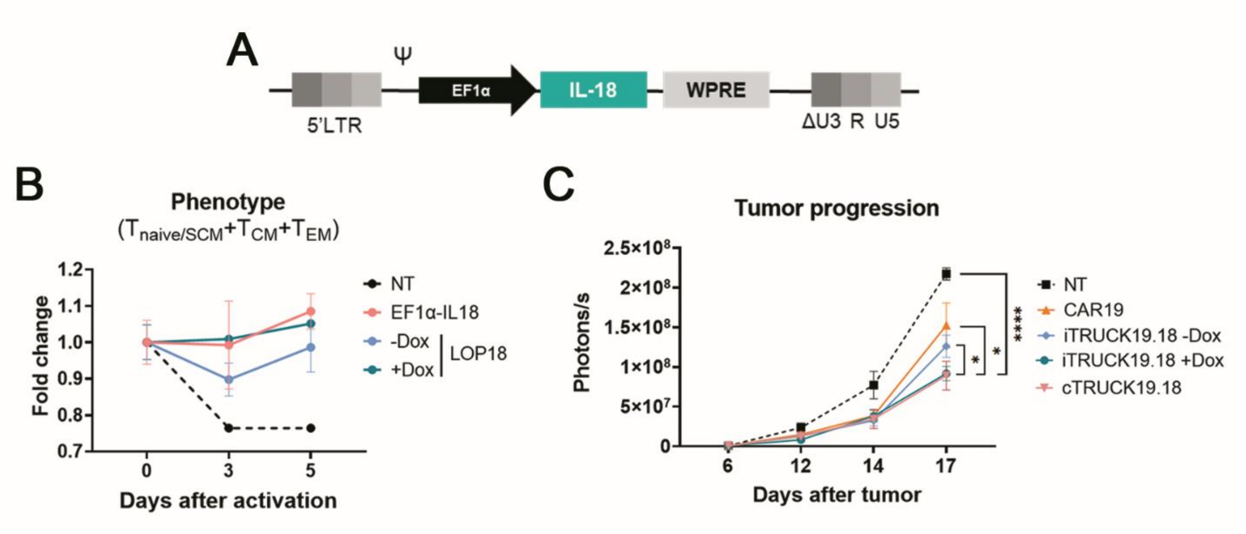

Fig S4. Phenotype and antitumor activity of CAR19 expressing IL-18 constitutively (cTRUCK19.18 cells). (A) Diagram of the lentiviral vector used for the generation of cTRUCK19.18. IL-18 is expressed under the control of EF1α promoter. (B) Phenotypic analysis of untransduced T cells (NT, black circles), cTRUCK19.18 (EF1α–IL18, orange circles), iTRUCK19.18 without Dox (LOP18 -Dox, ligh blue circles) and + 50ng/ml of Dox (LOP18 +Dox, dark blue circles) (NT: n=1; EF1α-IL-18, -Dox and +Dox: n=3). (C) Bioluminescence analysis of tumor progression *in vivo* using the Namalwa model. Once the tumor was established, mice were inoculated with $3x10^5$ CAR-T cells of CAR19 (orange triangles), cTRUCK19.18 (pink triangles), iTRUCK19.18 without Dox (light blue triangles) and in the presence of 1000ng/ml of Dox administered orally. Tumor progression was determined by bioluminescence (photons/s) of the different experimental group p<0.05, **p<0.01, ****p<0.0001 (one-tailed unpaired t test).

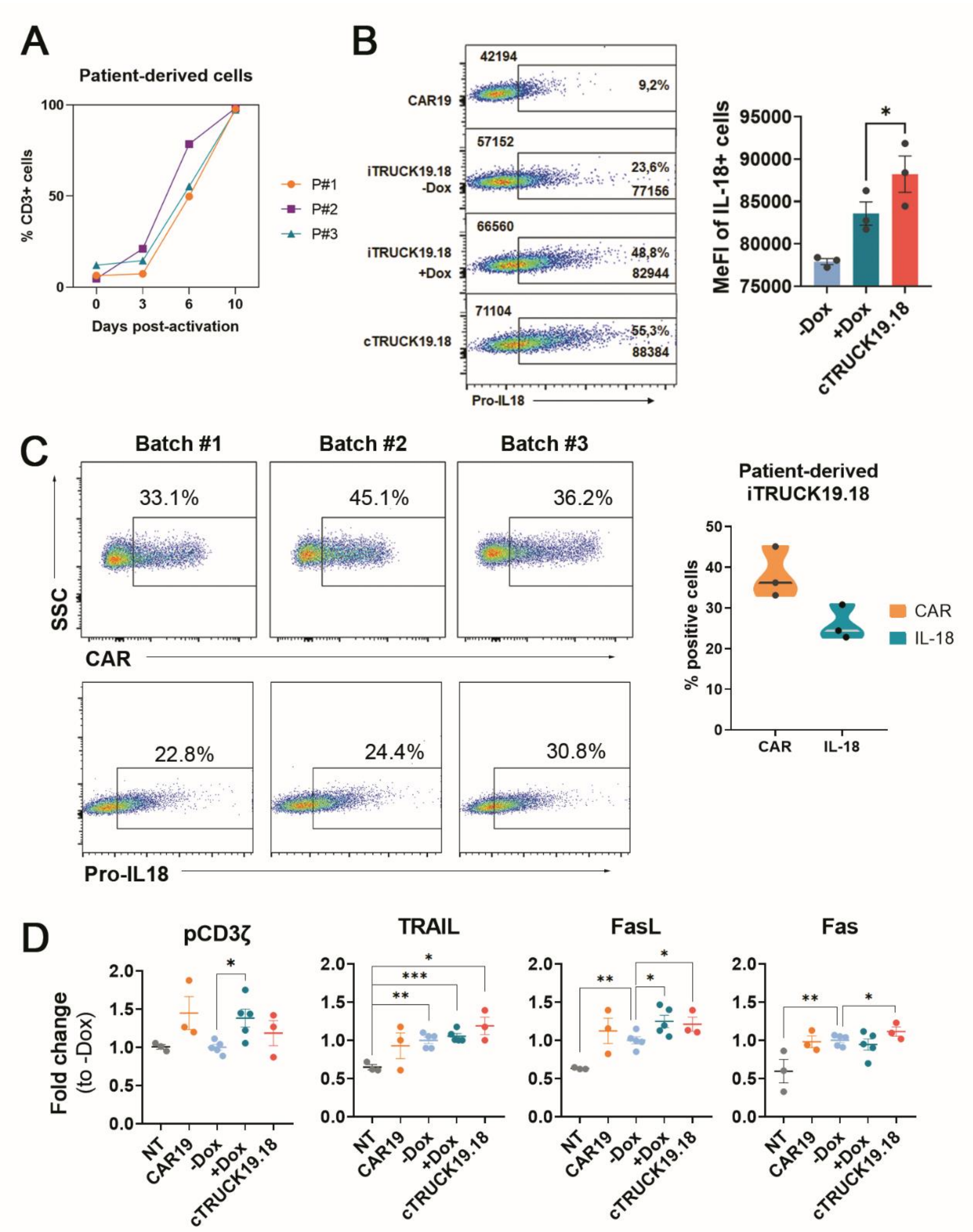

Fig. S5 Generation and characterization of patient-derived iTRUK19.18 cells. (A) Left- Enrichment of CD3+ cells from patient's #1, #2 and #3 after stimulation with TransAct during 6 days. (B) Left: representative dot-plots showing the pro-IL-18 expression levels of CAR19 (Top plot), cTRUCK19.18 (bottom plot), iTRUCK18.19 without Dox (second top plot) and iTRUCK18.19 with 50ng/ml of Dox (second bottom plot). Right. Graph showing the expression levels (as MeFI of IL-18+ cells) of cTRUCK19.18 (red bar), iTRUCK18.19 without Dox (light-

blue bar) and iTRUCK18.19 with 50ng/ml of Dox (dark-blue bar) (n=3). Paired t-test two-tails. *, $p<0.05$. (C) Left: Representative dot-plots showing CAR (top plots) and pro-IL-18 (bottom plots) expression levels of three different iTRUCK19.18 productions (batches 1,2,3) from three different patients with CD19+ malignances. Right: Graph showing variability of CAR (Orange) and pro-IL-18 (Green) expression levels of three different iTRUCK19.18 productions. All the data was collected in the presence of Dox at day 10 post-transduction (n=3). (D) Fold-change (relative to -Dox) of activation-related markers expression of patient-derived iTRUCK19.18 cells without and with (50 ng/ml). Dox treatment under basal conditions (from left to right: phospho-CD3ζ, TRAIL, FasL, and Fas). $p<0.05$, **$p<0.01$, ****$p<0.0001$ (one-tailed paired test).

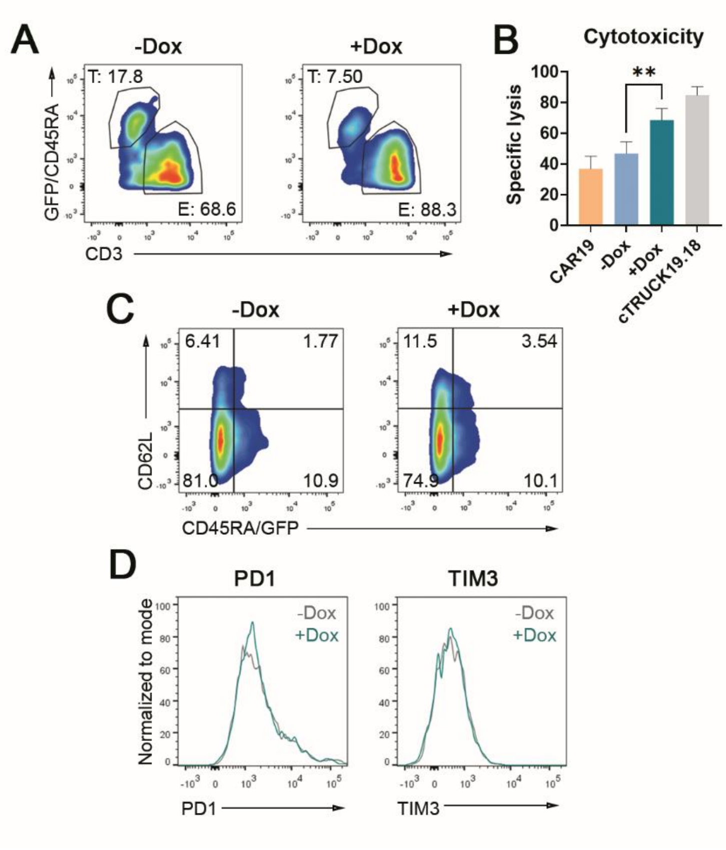

Fig. S6 Characterization of iTRUCK19.18 and cTRUCK19.18 cells. (A) Representative dot plots of iTRUCK19.18 cell lysis without (left) and with (50 ng/ml) (right) Dox from the first encounter with MIA-PaCa2 GFP-Nluc CD19+. (B) Specific lysis of CAR19, iTRUCK19.18 -Dox, iTRUCK19.18 +Dox and cTRUCK19.18 against MIA-PaCa2 GFP-Nluc at E:T ratio 1:2 (CAR19 and cTRUCK19.18: n=2; iTRUCK19.18 -Dox and +Dox: n=5. **p<0.01 (two tailed paired t test). (C) Representative dot plots of iTRUCK19.18 cell phenotype without (left) and with (50 ng/ml) (right) Dox. (D) Representative histograms of PD1 and TIM3 expression in iTRUCK19.18 cells without (left) and with (50 ng/ml) (right) Dox.

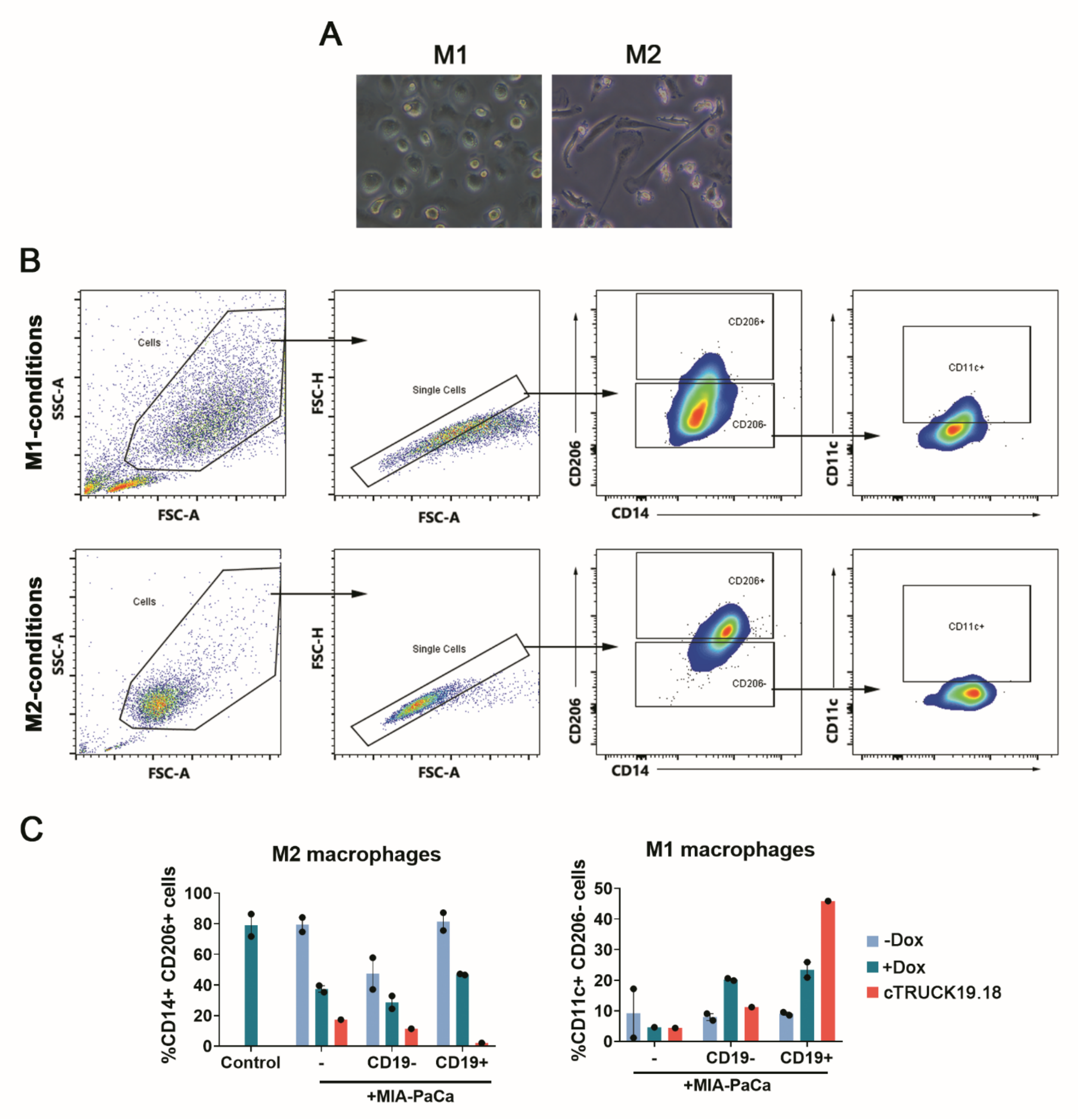

Fig. S7 Characterization of primary M1 and M2 macrophages and the effect of iTRUCK19.18 and cTRUCK19.18 on their polarization. (A) Bright-field microscopy images of human macrophages polarized to M1 (left) or M2 (right) phenotype. (B) Gating strategy for the identification of M1 and M2 macrophages by FACS according to CD14, CD206 and CD11c expression. (C) Percentage of M2 (Left; CD14+ CD206+ cells) and M1 (Right; CD11c+ cells) after the co-culture with iTRUCK19.18 (-Dox and +Dox, n=2) and cTRUCK19.18 cells (n=1) in

the absence of MIA-PACA (-) or in the presence of MIA-PaCA2-CD19 negative (CD19-) or of MIA-PaCA2-CD19+ (CD19+) during 6 days.
