# Supplementary material for: First-in-class transactivator-free, doxycycline-inducible IL-18-engineered CAR-T cells for relapsed/refractory B cell lymphomas
Source: Mol Ther Nucleic Acids. 2024 Aug 15;35(4):102308. doi: 10.1016/j.omtn.2024.102308 (PMC11617245; doi:10.1016/j.omtn.2024.102308)
Supplement: Document S2. Article plus supplemental information [file mmc2.pdf]

Molecular Therapy
Nucleic Acids
Original Article

First-in-class transactivator-free, doxycycline-inducible IL-18-engineered CAR-T cells for relapsed/refractory B cell lymphomas

Pedro Justicia-Lirio,[1,20] María Tristán-Manzano,[1,20,21] Noelia Maldonado-Pérez,[2,3] Carmen Barbero-Jiménez,[1] Marina Cortijo-Gutiérrez,[3] Kristina Pavlovic,[3,4] Francisco J. Molina-Estevez,[3,5] Pilar Muñoz,[3,5,6] Ana Hinckley-Boned,[3] Juan R. Rodriguez-Madoz,[7,8,9] Felipe Prosper,[7,8,9,10] Carmen Griñán-Lison,[5,11,12,13] Saúl A. Navarro-Marchal,[5,11,12,14] Carla Panisello,[15,16,17] Julia Muñoz-Ballester,[5,18] Pedro A. González-Sierra,[5,18] Concha Herrera,[4] Juan A. Marchal,[5,11,12,19] and Francisco Martín[2,3,5,11,21]

[1]LentiStem Biotech, Pfizer-University of Granada-Andalusian Regional Government Centre for Genomics and Oncological Research (GENYO), PTS, Av. de la Ilustración 114, 18016 Granada, Spain; [2]Department of Biochemistry and Molecular Biology III and Immunology, Faculty of Medicine, University of Granada, Av. de la Investigación, 11, 18006 Granada, Spain; [3]Department of Genomic Medicine, Pfizer-University of Granada-Andalusian Regional Government Centre for Genomics and Oncological Research (GENYO), PTS, Av. de la Ilustración 114, 18016 Granada, Spain; [4]Maimonides Institute of Biomedical Research in Cordoba (IMIBIC), Cellular Therapy Unit, Reina Sofía University Hospital, University of Cordoba, Av. Menéndez Pidal, 14004 Cordoba, Spain; [5]Instituto de Investigación Biosanitaria ibs.GRANADA, University Hospitals of Granada, University of Granada, Av. de Madrid 15, 18012 Granada, Spain; [6]Department of Cellular Biology, Faculty of Sciences, University of Granada, Av. de Fuente Nueva, 18071 Granada, Spain; [7]Centro de Investigacion Biomedica en Red de Cancer (CIBERONC), Madrid, Spain; [8]Hemato-Oncology Program, Cima Universidad de Navarra, IdiSNA, Pamplona, Spain; [9]Cancer Center Clinica Universidad de Navarra (CCUN), Pamplona, Spain; [10]Hematology and Cell Therapy Department, Clinica Universidad de Navarra, IdiSNA, Pamplona, Spain; [11]Excellence Research Unit "Modeling Nature" (MNat), University of Granada, 18016 Granada, Spain; [12]Biopathology and Regenerative Medicine Institute (IBIMER), Centre for Biomedical Research (CIBM), University of Granada, 18016 Granada, Spain; [13]Department of Biochemistry and Molecular Biology II, Faculty of Pharmacy, University of Granada, 18071 Granada, Spain; [14]Cancer Research UK Edinburgh Centre, Institute of Genetics and Cancer, University of Edinburgh, Edinburgh EH4 2XU, UK; [15]Josep Carreras Leukemia Research Institute, Barcelona, Spain; [16]Germans Trias i Pujol Research Institute (IGTP), Badalona, Spain; [17]Red Española de Terapias Avanzadas (TERAV)-Instituto de Salud Carlos III (ISCIII), Madrid, Spain; [18]Hematology and Hemotherapy Unit, Virgen de las Nieves University Hospital, Av. de las Fuerzas Armadas 2, 18014 Granada, Spain; [19]Department of Human Anatomy and Embryology, Faculty of Medicine, University of Granada, Av. de la Investigación 11, 18006 Granada, Spain

Although chimeric antigen receptor (CAR) T cell therapy has revolutionized type B cancer treatment, efficacy remains limited in various lymphomas and solid tumors. Reinforcing conventional CAR-T cells to release cytokines can improve their efficacy but also increase safety concerns. Several strategies have been developed to regulate their secretion using minimal promoters that are controlled by chimeric proteins harboring transactivators. However, these chimeric proteins can disrupt the normal physiology of T cells. Here, we present the first transactivator-free anti-CD19 CAR-T cells able to control IL-18 expression (iTRUCK19.18) under ultra-low doses of doxycycline and without altering cellular fitness. Interestingly, IL-18 secretion requires T cell activation in addition to doxycycline, allowing the external regulation of CAR-T cell potency. This effect was translated into an increased CAR-T cell antitumor activity against aggressive hematologic and solid tumor models. In a clinically relevant context, we generated patient-derived iTRUCK19.18 cells capable of eradicating primary B cells tumors in a doxycycline-dependent manner. Furthermore, IL-18-releasing CAR-T cells polarized pro-tumoral macrophages toward an antitumoral phenotype, suggesting potential for modulating the tumor microenvironment. In summary, we showed that our platform can generate exogenously controlled CAR-T cells with enhanced potency and in the absence of transactivators.

INTRODUCTION

CD19-redirected chimeric antigen receptor (CAR)-T cell therapy has provided long-lasting clinical responses treating relapsed and/or refractory B cell neoplasms.[1] The high complete remission rate achieved in patients unresponsive to multiple lines of treatment has led the FDA and EMA to approve six CAR-T-based advanced therapy medicinal products (ATMPs) to date.

Received 26 February 2024; accepted 12 August 2024;
https://doi.org/10.1016/j.omtn.2024.102308.

[20]These authors contributed equally
[21]Senior author
Correspondence: María Tristán-Manzano, LentiStem Biotech, Pfizer-University of Granada-Andalusian Regional Government Centre for Genomics and Oncological Research (GENYO), PTS, Av. de la Ilustración 114, 18016 Granada, Spain.
E-mail: mtristan@lentistem.com
Correspondence: Francisco Martín, Department of Biochemistry and Molecular Biology III and Immunology, Faculty of Medicine, University of Granada, Av. de la Investigación, 11, 18006 Granada, Spain.
E-mail: francisco.martin@genyo.es

Published by Elsevier Inc. on behalf of The American Society of Gene and Cell Therapy.
This is an open access article under the CC BY-NC-ND license (http://creativecommons.org/licenses/by-nc-nd/4.0/).

Despite outstanding results in the treatment of different CD19+ hematologic cancers, around 30%–50% of patients relapse after αCD19-CAR-T infusion.[2] CAR-T therapy has shown limited therapeutic efficacy in other hematological malignancies such as chronic lymphocytic leukemia (CLL),[3] and very few reports have shown efficacy on solid tumors.[4] Several factors appear to be influencing the loss of CAR-T efficacy: (1) CAR-derived tonic signaling, which accelerates functional exhaustion and toxicity of CAR-T cells,[5] (2) impaired long-term CAR-T cell persistence,[6] (3) restricted trafficking of CAR-T cells into the tumor,[7] and (4) a highly immunosuppressive tumor microenvironment (TME).

One of the possible solutions to improve the therapeutic efficacy of CAR-T involves the fine-tuning of TRUCKs (T cells redirected for antigen-unrestricted cytokine-initiated killing), CAR-T cells engineered to release transgenic proteins that boost their antitumor capacity. Generally, TRUCKs co-express interleukins such as IL-7, IL-12, IL-15, or IL-18. These key cytokines exert an immunomodulatory role in reversing the pro-tumor environment of the TME into an antitumor milieu, promoting a coordinated immune cell attack on the tumor.[8] However, the expression of these cytokines in long-lived T cells requires tight regulation to avoid a plethora of potential unwanted side effects. Up to date, most TRUCKs designs use endogenously regulated NFAT (nuclear factor of activated T cell)-based promoters to express cytokines to generate tumor-specific T cells that secrete the selected cytokine only after T cell activation.[9] However, several studies suggest the inability of the NFAT promoter to efficiently control cytokine expression inside the tumor.[10,11] This likely arises from the presence of multiple signals beyond just CAR or TCR binding to its target, which can initiate NFAT activation. These signals include G protein-coupled receptor signaling (involved in T cell migration and activation),[12] proinflammatory cytokines (IL-2, IL-4, IL-6, and TNF-α),[13] and viral infections.[14] Therefore, regulating T cell potency by NFAT-driven promoters remains challenging. Alsaieedi et al. discovered that, while the expression of IL-12 was necessary to observe antitumor effectiveness, the introduction of NFAT-IL-12 transgenic T cells into a syngeneic murine model of B16F10 melanoma led to lethality.[11] In the same direction and in a clinical context, Zhang et al. observed severe toxicity in melanoma patients treated with autologous tumor-infiltrating lymphocytes (TILs) genetically engineered to express IL-12 under NFAT promoter.[10]

As an alternative to endogenously regulated NFAT-driven promoters, several groups are developing exogenously inducible promoters,[15] such as the one based on the bacterial TetO operon. In this direction Alsaieedi et al. showed that, while NFAT-driven IL-12-engineered T cells induce lethality (see above), Tet-On-engineered T cells were safe in the absence of doxycycline (Dox) and that temporal induction of IL-12 inhibits the growth of B16F10 melanoma tumors. These data demonstrate the potency of Dox-inducible Tet-On systems as tools to generate smart ATMPs that can be controlled externally by clinicians. However, most Tet-On systems require a transactivator (a chimeric protein composed of the bacterial Tet repressor [TetR] and the activating domain of the viral protein 16 of herpes simplex virus type 1) to achieve inducibility. These transactivators showed multiple side effects on gene-modified cells due to transcription factor sequestering or by binding to pseudo TetO sites.[16–19] Importantly, Smith et al. observed a depletion of antigen-experienced T cells in reverse tetracycline-controlled transactivator (rtTA)-transgenic mice, demonstrating the potential difficulties of using these systems to generate clinical-grade inducible T cells. To tackle these problems, our group has previously developed insulated,[20] transactivator-free, Tet-On lentiviral vectors (LVs) (Lent-On-Plus or LOP)[21,22] that tightly regulate transgene expression in a variety of primary human cells, including T cells, without altering physiology and using ultra-low doses of Dox.[23]

Previous studies have shown that constitutive expression of IL-18 by CAR-T cells significantly enhances the antitumor activity of CAR-T cells. IL-18 is a cytokine of the IL-1 family constitutively produced by activated macrophages, and dendritic and epithelial cells. It directly stimulates interferon gamma (IFN-γ) secretion and other inflammatory cytokines and chemokines, exhibiting pleiotropic effects on the entire immune system via Th1 immune response.[24] IL-18 enhances the cytotoxic activity of T cells and natural killer (NK) cells by upregulation of Fas ligand (FasL),[25] polarization of pro-tumorigenic M2 to antitumor M1 macrophages,[26] and by acting in synergy with other cytokines.[27] Recently, it was reported that IL-18-secreting CAR-T cells showed superior antitumor activity via the helper effect of CD4+ CAR-T cells for the augmentation of CD8+ CAR-T cells.[28] Avanzi et al. demonstrated that IL-18 CAR-T cells were able to eliminate liquid and solid tumors in syngeneic murine models[29] Taking all these data, the University of Pennsylvania's team is currently running a clinical trial (NCT04684563, phase 1) co-expressing IL-18 on αCD19-CAR-T cells to evaluate the maximum safe dose.[30]

As for most cytokines, uncontrolled release of IL-18 can lead to potential safety issues, since a continuous delivery of IL-18 promotes constant (and non-tissue specific) IFN-γ secretion, creating a permanent environment of acute inflammation that might lead to toxicity[31] and autoimmune disorders,[32,33] as well as IFN-γ-independent toxicities.[34] IL-18 can potentially trigger ICANS (immune effector cell-associated neurotoxicity syndrome) in CD19+ therapy[35] or associated hemophagocytic lymphohistiocytosis-like toxicity.[36] Although no serious effects have been described so far in the clinical trial mentioned above,[30] the potential toxicities associated with unregulated expression of IL-18 remain a concern. Therefore, even though constitutive high expression of IL-18 can lead to greater anti-tumor activity compared with inducible systems, the ability to control IL-18 secretion should still be considered a safer alternative for enhancing the anti-tumor activity of CAR-T cells. In this regard, Chmielewski et al. generated CAR-T cells able to release IL-18 "on demand" using an NFAT promoter. The authors showed that their engineered CAR-T cells released IL-18 in a CAR-dependent fashion and increased the antitumor effect compared with standard CAR-T cells[26] However, as outlined above, several studies suggest the inability of the NFAT promoter to efficiently control cytokine expression *in vivo*.[10,11]

As mentioned before, Dox-based inducible expression systems have emerged as interesting alternatives to control the expression of

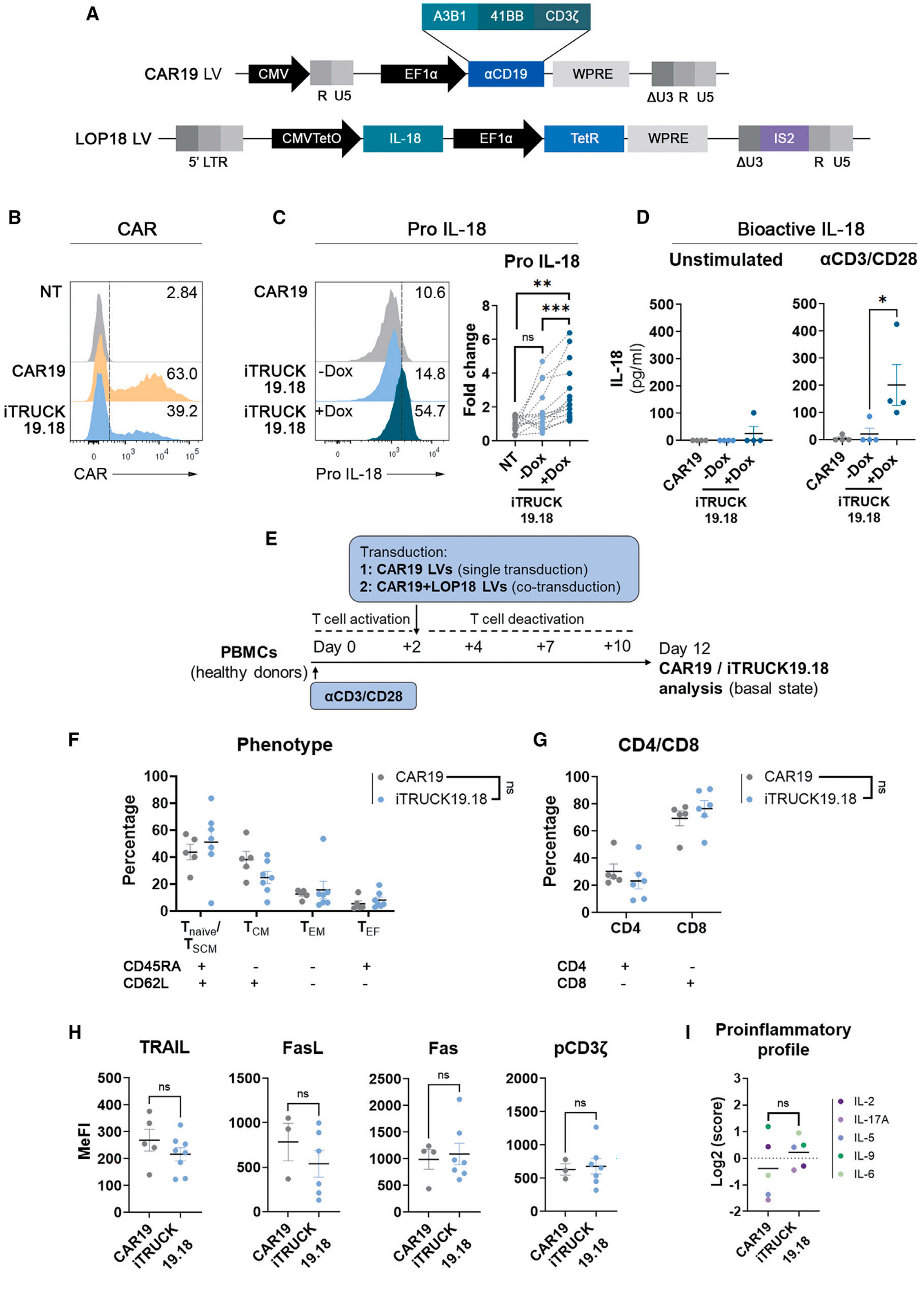

(legend on next page)

cytokines with the limitation of high Dox requirements and the presence of highly toxic transactivators. Here, we describe *first-in-class* αCD19-CAR-T cells (iTRUCK19.18) engineered to release IL-18 under ultra-low (subtherapeutic) Dox doses and in the absence of transactivators. iTRUCK19.18 controlled IL-18 expression both *in vitro* and *in vivo*, allowing the control of T cell potency and polarizing pro-tumoral M2 macrophages toward an antitumoral phenotype (M1) in a Dox-dependent manner. This effect was translated into an increased CAR-T cell antitumor activity against an aggressive hematologic and an engineered CD19+ pancreatic ductal adenocarcinoma (PDAC) model. In a clinically relevant context, we also generated patient-derived iTRUCK19.18 and observed that the Dox-dependent release of IL-18 improved the eradication of primary B cell tumors.

RESULTS

Generation of transactivator-free, IL-18-inducible αCD19 CAR-T cells (iTRUCK19.18)

As recently described by our group, LOP LVs can tightly regulate transgene expression in primary T cells *in vitro* and *in vivo*.[23] Based on these data, we decided to generate CAR-T cells that express IL-18 in an inducible manner (iTRUCK19.18) using the LOP system. To achieve this, we co-transduced primary T cells with CAR19 LVs (multiplicity of infection [MOI] = 3) (allowing constitutive expression of a 4-1BB αCD19 CAR endowed with the A3B1 scFv clone)[37] (Figure 1A, top) and LOP18 (MOI = 5) (for Dox-inducible expression of IL-18 using the LOP LV) (Figure 1A, bottom), resulting in a heterogeneous population of cells including CAR+IL-18+, CAR+IL-18−, CAR−IL-18+, and CAR−IL-18− cells (Figure S1A). Co-transduction with two LVs seems to reduce CAR expression compared with transduction with single CAR19 LVs (Figures 1B and S1B, left), probably due to LV dilution and free VSV-G protein competing for free receptors. We achieved ∼30% CAR and ∼20% IL-18 among different batches (Figure S1C). Importantly, 7–9 days post-transduction, iTRUCK19.18 showed minimal intracellular pro-IL-18 expression in the absence of Dox (Figure 1C, left, blue histogram; Figure 1C, right, blue dots) and up to 44.1% in its presence (Figure 1C, left, green histogram; Figure 1C, right, green dots; Figure S1B, right). Of note, the system required 7–9 days to achieve complete regulation, probably due to the requirement to achieve enough TetR protein concentrations in the nuclei to block transcription (data not shown).[38] We also showed that mRNA IL-18 levels were reduced to near baseline levels after 8 days post Dox (Figure S1A).

Once we confirmed that iTRUCK19.18 cells induced pro-IL-18 in a Dox-dependent manner, our subsequent aim was to verify the accurate processing and secretion of the cytokine. Physiologically, IL-18 is synthesized as a pro-peptide mainly by activated macrophages, dendritic and epithelial cells. Activation signal or tissue damage triggers the pro-caspase-1 processing into functional caspase-1 by the inflammasome complex, converting pro-IL-18 into mature IL-18, which is secreted.[39] We therefore analyzed if transgenic IL-18 expressed by iTRUCK19.18 cells follows a similar process despite being expressed in a non-natural context. Our findings are consistent with this mechanism, as iTRUCK19.18 cells necessitate T cell stimulation in conjunction with the presence of Dox to secrete bioactive IL-18 (Figure 1D). This observation aligns with the natural processing of IL-18, endowing these cells with a dual switch mechanism involving both T cell activation and Dox exposure. This configuration enhances their safety profile.

With the aim of characterizing the generated product, we conducted a comparative analysis of iTRUCK19.18 cells and CAR19 cells to decipher whether co-transduction affected production and T cell fitness compared with the generation of conventional CAR-T cells. We analyzed the immunophenotype (following the gating strategy showed on Figure S1B), CD4/CD8 cell ratio, activation-induced cell death (AICD) markers, signaling through CD3ζ phosphorylation (pCD3ζ), and the proinflammatory profile by measuring the secretion of five proinflammatory cytokines at the end of the production process (12 days, in basal state) (Figure 1E). We found no significant differences between iTRUCK19.18 cells and CAR19 cells in any of the parameters analyzed (Figures 1F–1I), indicating the feasibility of generating iTRUCK19.18 by co-transduction with CAR19 and LOP18 LVs (Figures S1A and S1C). However, we must consider that, on average, only 20% of the T cells express IL-18 and therefore the potential of this cytokine can be underestimated.

Dox addition to iTRUCK19.18 cells enhanced their activation capacity without compromising T cell exhaustion and phenotype

Once it was confirmed that the production process of iTRUCK19.18 cells was feasible, we analyzed the effect of IL-18 production on T cells

Figure 1. Generation and characterization of inducible IL-18-producing CAR-T cells (iTRUCK19.18)

(A) CAR19 LV encoding for EF1α-A3B1-41BB-CD3ζ (top) and Dox-inducible LOP LVs expressing pro-IL-18 (bottom). (B) Representative histograms of CAR expression in non-transduced cells (NT) (top), CAR19 (middle), and iTRUCK19.18 −Dox (bottom). (C) Representative histograms of pro-IL-18 expression in CAR19 cells (top) and iTRUCK19.18 cells in the absence (middle) or presence (bottom) of 50 ng/mL Dox (48 h). Right: fold change of IL-18 expression from iTRUCK19.18 cells relative to basal background of NT ($n = 17$; −Dox: $n = 15$; +Dox: $n = 17$) (right). (D) Bioactive IL-18 secreted by iTRUCK19.18 without activation (left) and activating with TransAct (αCD3/CD28) (right) ($n = 4$). (E) Experimental procedure for CAR19 and iTRUCK19.18 cell generation (single or co-transduction with LVs) and their analysis in resting conditions after 10 days in the absence of stimuli (without Dox). (F) Phenotype of CAR19 and iTRUCK19.18 cells in the absence of Dox. Total T cells (CD3+) were analyzed for CD45RA and CD62L expression (CAR19 cells: $n = 5$; iTRUCK19.18: $n = 7$). (G) CD4/CD8 ratio of CAR19 and iTRUCK19.18 cells in the absence of Dox of the total populations (CD3+) (CAR19 cells: $n = 5$; iTRUCK19.18: $n = 7$). (H) Expression of AICD markers (TRAIL, FasL, Fas) and phosphorylation of CD3z in CAR19 vs. iTRUCK19.18 cells (−Dox) in CD3+ cells: TRAIL (CAR19 T cells: $n = 5$; iTRUCK19.18: $n = 8$), FasL (CAR19 T cells: $n = 3$; iTRUCK19.18: $n = 6$), Fas (CAR19 T cells: $n = 4$; iTRUCK19.18: $n = 7$), pCD3ζ (CAR19 T cells: $n = 3$; iTRUCK19.18: $n = 7$). (I) Index of proinflammatory cytokine-related secretion by CAR19 T cells vs. iTRUCK19.18 cells at basal state ($n = 2$). *$p < 0.05$, **$p < 0.01$, ***$p < 0.001$ (two-tailed paired t test).

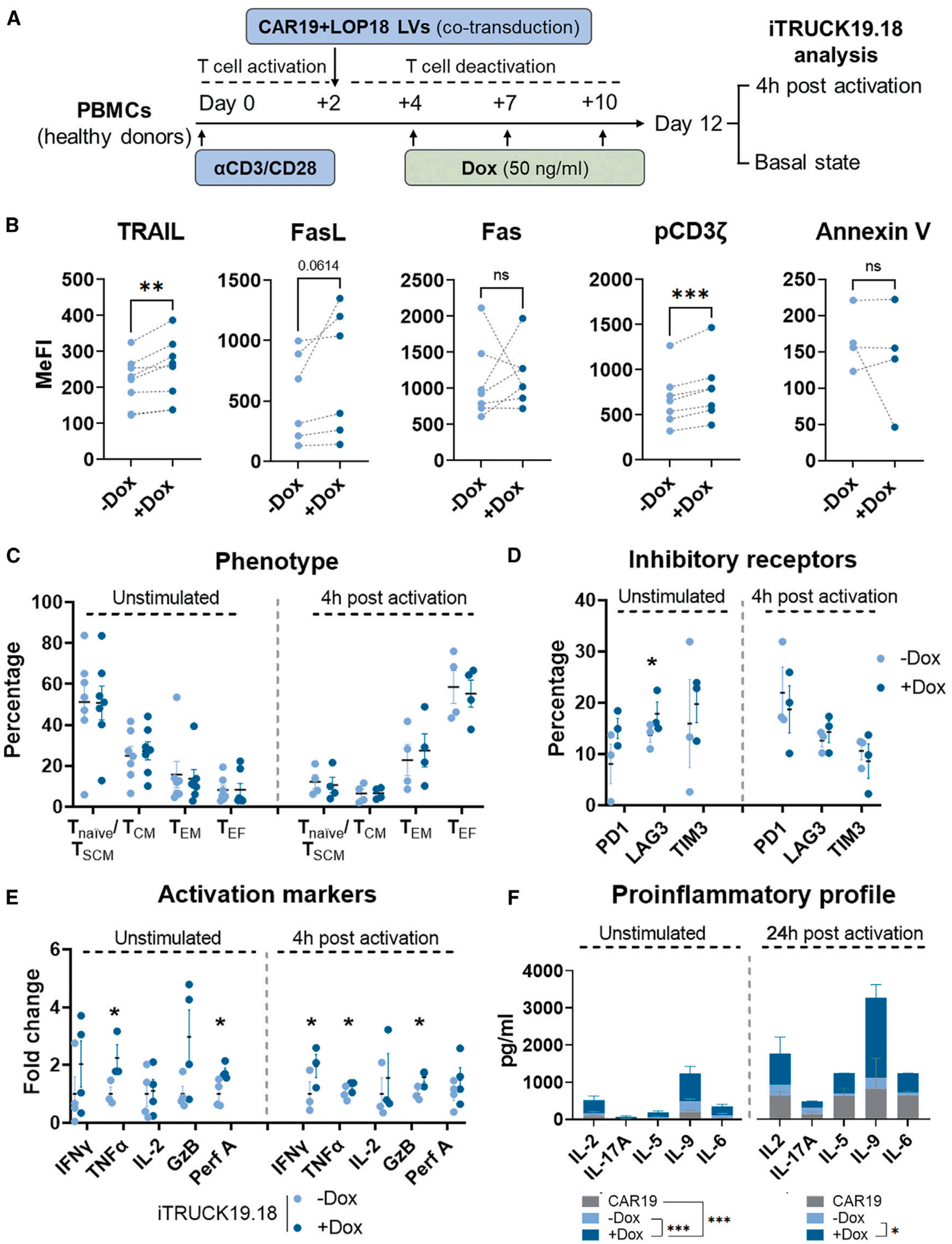

Figure 2. Characterization of iTRUCK19.18 cells in absence and presence of Dox

(A) Scheme for iTRUCK19.18 cell generation and analysis at basal state or after activation with αCD3/CD28. (B) Expression of AICD markers (TRAIL, FasL, Fas), phosphorylation of CD3z and apoptosis marker Annexin V (from left to right) in iTRUCK19.18 cells at basal state in the absence (light blue) or presence of 50 ng/mL Dox (dark blue): TRAIL ($n = 7$), FasL ($n = 6$), Fas ($n = 7$), pCD3ζ ($n = 7$), and Annexin V ($n = 4$). Analysis performed on total CD3+ T cells. (C) Phenotype of iTRUCK19.18 cells with (dark blue) and without Dox (light blue) at resting conditions (left) ($n = 7$) or after 4 h of stimulation (right) ($n = 4$). Analysis performed on total CD3+ T cells. (D) Percentage of positive cells in the

(legend continued on next page)

(Figure 2A). IL-18 plays a pivotal role in the activation of T cells, so initially we evaluated the effect of IL-18 production at basal level.

To evaluate the effect on AICD, we analyzed TNF-related apoptosis-inducing ligand (TRAIL), FasL, and Fas expression in total CD3+ (CAR+ and CAR−) iTRUCK19.18 cells under both Dox-induced and non-induced conditions. Upon Dox exposure, we observed an upregulation of TRAIL (Figure 2B, first graph), a tendency in FasL (Figure 2B, second graph), but no difference was observed in Fas expression (Figure 2B, third graph). Interestingly, Dox administration led to a significant increase in the expression of phospho-CD3ζ (Figure 2B, fourth graph) (representative dot plots on Figure S2A). However, despite the notable changes in AICD and tonic signaling, we did not observe any differences in apoptosis in the presence of Dox (Figure 2B, right graph).

Next, we aimed to conduct a more comprehensive characterization of the fitness of iTRUCK19.18 cells, not only at the basal level but also after stimulation. At a basal level, IL-18 production did not alter the ratio of CD4+/CD8+ T cells (Figure S2B) or the distribution of phenotypic subpopulations on total CD3+ cells (Figure 2C). In addition, the expression of inhibitory receptors PD1, LAG3, and TIM3 on T cells remained unaffected, except for a significant increase in the expression of LAG3 in the basal state. However, upon activation, we observed that this increase in LAG3 expression did not persist (Figure 2D). This suggests that the production of this cytokine does not accelerate T cell exhaustion. We next analyze a panel of activation markers and proinflammatory profile in unstimulated and CD3/CD28-stimulated iTRUCK19.18 cells in the presence or absence of Dox on total CD3+ cells (Figure 2E). As expected, the expression of IL-18 in iTRUCK19.18 cells upon Dox addition increased the expression of several activation markers. Indeed, following cellular activation, we observed a significant upregulation in the expression of IFN-γ, TNF-α, and Granzyme B, along with a trend toward higher expression of IL-2 and Perforin A. Interestingly, Perforin A and TNF-α were significantly increased under basal conditions in the presence of Dox (Figure 2E). The increased activation state of iTRUCK19.18 cells in response to Dox is consistent with the augmentation of their proinflammatory profile, as evidenced by the upregulated secretion of IL-2, IL-17A, IL-5, IL-9, and IL-6 (Figures 2F and S2C). Of note, we also observed a trend in the increase of IL-4 and IL-10 (Figure S2D), underlining the dual role of the cytokine depending on the context.

It is important to notice that no significant differences were found in single transduced CAR19 cells (without LOP18) by the use of Dox in any of the markers analyzed either at basal state or after activation (Figures S3A–S3E), indicating that the observed effects are exclusively due to the production of IL-18 and not by the Dox per se. Considering all these data, we conclude that Dox addition increased the activation of iTRUCK19.18 cells while retaining the exhaustion state without significant phenotypic alterations.

DOX regulates the antitumoral activity of iTRUCK19.18 cells in a Burkitt lymphoma model *in vitro* and *in vivo*

Once demonstrated that the secretion of functional IL-18 by iTRUCK19.18 cells can be controlled by Dox, maintaining an appropriate phenotype, we analyzed whether we can also control their antitumoral activity. For this purpose, we first co-cultured iTRUCK19.18 and CAR19 cells with Namalwa cells, a Burkitt lymphoma cell model, using serial tumor stimulations (Figure 3A). The results demonstrated that, during the third tumor encounter, IL-18-releasing iTRUCK19.18 cells exhibited a significant enhanced antitumoral activity compared with cells without Dox and standard CAR19 cells. Furthermore, even the Dox-free condition displayed greater antitumoral action than CAR19 cells, suggesting that the initial secretion of IL-18 during the initial days post-transduction (the system requires 6–10 days post-transduction to achieve tight regulation) is having a positive effect on their fitness/antitumoral activity (Figure 3B).

Next, we assessed the *in vivo* efficacy of iTRUCK19.18 cells. For this purpose, 0.3×10^6 Namalwa green fluorescent protein-nanoluciferase (GFP-Nluc) cells were infused into immunocompromised NOD/scid-IL-2Rnull mice (NSG) mice and tumor progression was monitored after intravenous administration of 1×10^6 iTRUCK19.18 cells (in the presence or absence of Dox), CAR19 cells, non-transduced T cells (NT) and PBS (Figure 3C). After seven days of CAR-T cells administration, we observed a significant increase in human circulating T cells in the blood of mice treated with iTRUCK19.18 only under oral Dox supplementation (Figure 3D, left). Furthermore, we observed that human T cells from mice treated with Dox significantly induced IL-18 expression (Figure 3D, right), demonstrating that oral administration of Dox allows *in vivo* induction of IL-18.

In this context (with a high quantity of CAR-T cells and a low tumor burden), no differences in antitumoral response were observed between the different groups, as all mice treated with CAR19/iTRUCK19.18 completely eradicated lymphoma even after two additional tumor re-infusions (re-challenge 1 and 2, R1 and R2) (Figures 3E and 3F). Interestingly, the analysis of tumor cell infiltration in different tissues showed that iTRUCK19.18-treated mice completely cleared tumor cells in all tissues in the absence or presence of Dox, while 1/5 CAR19 cell-treated mouse presented brain metastasis (Figure 3G). Unfortunately, we stopped the experiment on day

CD3+ population for exhaustion markers PD1, LAG3, TIM3 of iTRUCK19.18 cells at basal state and after stimulation in the absence or presence of 50 ng/mL Dox (*n* = 4). (E) Fold change was calculated by dividing the percentage of +Dox population by the −Dox population (% at +Dox/% −Dox) for each of the activation markers analyzed (IFN-γ, TNF-α, IL-2, Granzyme B, and Perforin A) in iTRUCK19.18 cells, both without Dox (light blue) and with Dox (dark blue), at resting state (left) or after activation (right) (*n* = 4). (F) Secretion of proinflammatory cytokines from CAR19 (gray) and iTRUCK19.18 cells with (dark green) and without (light blue) Dox at basal state (*n* = 4) and after 24 h of stimulation with TransAct. Two-way ANOVA, multiple comparison Tukey's test. *$p < 0.05$, ***$p < 0.001$.

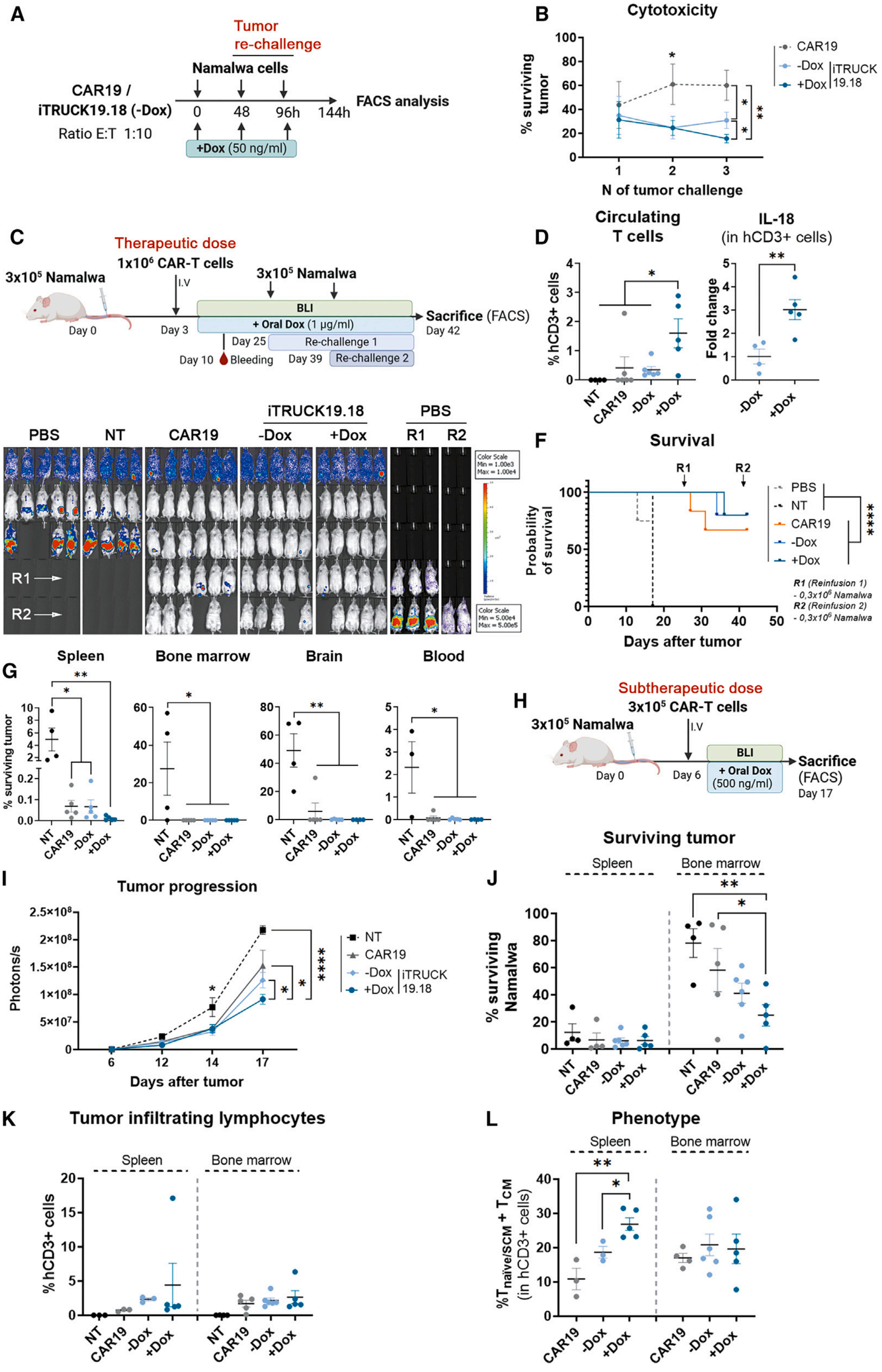

(legend on next page)

42 due to the development of xenogeneic graft-versus-host-disease (xenoGVHD).

Since the high CAR-T cell dose prevented from seeing differences in antitumor potency, we performed a new *in vivo* experiment where we infused a subtherapeutic dose (0.3 $\times$ 10^6 CAR-T cells/mouse) to mice under a higher tumor burden (allowing tumor expansion 6 days) (Figure 3H). In this new scenario, we did observe how the addition of Dox increased the antitumor potency of iTRUCK19.18 cells compared with iTRUCK19.18 −Dox-, CAR19-, and NT-treated mice (Figure 3I). Furthermore, as control, we generated CAR-T cells that constitutively express IL-18 (referred to as cTRUCK19.18) using LV EF1α-IL-18 (depicted in Figure S4A). No phenotypic changes were observed between cTRUCK19.18 and iTRUCK19.18 with Dox (Figure S4B). Similarly, *in vivo* administration of cTRUCK19.18 was equally efficient compared with iTRUCK19.18 with Dox in terms of tumor progression (Figure S4B). On day 17 we stopped the experiment (due to systemic progression of the lymphoma), and we analyzed the presence of surviving tumor cells, and the phenotype of the infiltrated T cells in the spleen and bone marrow. No significant differences were found in terms of infiltrated tumor cells (surviving Namalwa) among the groups in spleen (Figure 3J, left). However, mice treated with iTRUCK19.18 cells in the presence of Dox significantly reduced tumor cell invasion in bone marrow (Figure 3J, right). No significant differences were observed in the infiltration of human T cells in the spleen and bone marrow among the different groups of mice treated with CAR-T cells (Figure 3K). Furthermore, in the spleen of mice treated with iTRUCK19.18 +Dox, the infiltrated T cells exhibited a less differentiated/more memory phenotype (CD45RA+ CD62L+ and CD45RA− CD62L+) (Figure 3L, left), which might have contributed to the enhanced antitumoral efficacy. However, no discernible differences were observed in the bone marrow concerning the phenotype of mice infused with CAR-T cells (Figure 3L, right).

Healthy donor and patient-derived iTRUCK19.18 cells show an increased antitumor potency against primary B-type tumors in the presence of Dox

After confirming that the production of IL-18 by CAR-T cells enhanced the antitumoral effect against a Burkitt lymphoma model, we wanted to validate the use of iTRUCK19.18 cells in a clinically relevant setting. We isolated primary tumors and peripheral blood mononuclear cells (PBMCs) from three patients with B cell neoplasms expressing heterogeneous levels of CD19 (patient 1 diagnosed with marginal zone lymphoma [MZL] and patients 2 and 3 with CLL), (Figure 4A). We generated CD19 CAR-T cells, iTRUCK19.18 cells, and cTRUCK19.18 cells from patient PBMCs stimulated and enriched in CD3+ at the moment of the transduction (Figure S5A) by transduction with CAR19 LVs, co-transduction with CAR19 LVs and LOP18 LVs, and co-transduction with CAR19 LVs and EF1α-IL-18 LVs, respectively, as depicted in Figure 4B. CAR expression levels between 20% and 50% were achieved in all cases (Figure 4C). As expected, the addition of Dox resulted in the induction of pro-IL-18 (Figures 4D and S5B). In this line, we have obtained ∼38% of CAR+ cells and ∼26% expressing IL-18 (Figure S5C). We next assessed pCD3ζ, TRAIL, FasL, and Fas in patient-derived CD19 CAR-T cells, iTRUCK19.18 cells (+/− Dox), and cTRUCK19.18 cells. We found that Dox addition increased activation (pCD3ζ and FasL) of patient-derived iTRUCK19.18 cells (Figure S5D), consistent with our previous observations (Figure 2B). No differences were observed between iTRUCK19.18 cells +Dox and cTRUCK19.18 cells (Figure S5D).

Finally, in an autologous setting, we corroborated that patient-derived iTRUCK19.18 cells exhibited enhanced efficacy in eliminating the same-patient tumor B cells only when IL-18 is induced by Dox (Figure 4E, second-right bar). Interestingly, cTRUCK19.18 cells showed similar antitumor efficacy compared with iTRUCK19.18 cells in the presence of Dox Figure 4E, right bar). In addition, we found that IL-18-expressing cells, including iTRUCK19.18 (+Dox) and cTRUCK19.18 cells, reduce the expression transcription factor Tox (Figure 4F), which was associated with exhaustion and senescence in T cells. These compelling findings underscore the therapeutic potential of iTRUCK19.18 cells in treating B-type hematological neoplasms.

Dox treatment on iTRUCK19.18 cells increases their antitumoral potency against metastatic CD19+ PDAC model

Based on the results obtained from applying iTRUCK19.18 cells to hematologic cancer models, we aimed to investigate potential future

Figure 3. *In vitro* and *in vivo* evaluation of iTRUCK19.18 cells against B cell lymphoma model

(A) Diagram of *in vitro* cytotoxicity assay: 50 ng/mL of Dox was added at the moment of the co-culture and the dose was refreshed in every challenge. (B) Percentage of surviving Namalwa cells after serial tumor encounters with CAR19 or iTRUCK19.18 cells without and with Dox (n = 4). (C) *In vivo* experimental procedure to evaluate iTRUCK19.18 at a therapeutic dose (1 $\times$ 10^6 CAR-T cells/mouse). Dox (1,000 ng/mL) was added to drinking water after infusion into mice and refreshed twice a week. Two more tumor challenges with Namalwa cells were infused at days 25 and 42, respectively. (D) Proportion of circulating human T cells (left) and relative expression of IL-18 (right) by T cells obtained from blood 7 days after infusion of iTRUCK19.18 cells. (E) Bioluminescence images of tumor progression in mice treated with PBS, NT, CAR19, and iTRUCK19.18 without and with Dox. As control of re-challenges 1 and 2 (R1 and R2), novel mice were also infused with PBS at days +25 and +39. (F) Survival graph of mice treated with PBS, NT, CAR19, and iTRUCK19.18 without (−Dox, blue line) and with (+Dox, green line) Dox. (G) Percentage of viable tumor cells in different organs (spleen, bone marrow, brain, and blood, from left to right) of mice treated with NT, CAR19, and iTRUCK19.18 without (−Dox) and with Dox (+Dox), at final point (PBS: N = 5; NT: N = 4; CAR19: N = 6; iTRUCK19.18 −Dox: N = 5; iTRUCK19.18 +Dox: N = 5). (H) Diagram representing the infusion of a subtherapeutic dose (3 $\times$ 10^5 CAR-T cells) into mice 6 days post-tumor. Dox (500 ng/mL) was added to drinking water after the infusion of the CAR-T cells into mice and was refreshed twice a week. (I) Tumor progression determined by bioluminescence (photons/s) of the different experimental groups (NT, CAR19, iTRUCK19.18 −Dox, and iTRUCK19.18 +Dox. (J) Percentage of surviving tumor cells in the spleen (left) and bone marrow (right) of the mice from the different experimental groups. (K) Percentage of tumor-infiltrated T cells (hCD3+) in spleen (left) and bone marrow (right) of mice at the time of sacrifice. (L) Proportion of $T_{naive/SCM+TCM}$ cells in the spleen (left) bone marrow (right) of mice at endpoint (NT: N = 4; CAR19: N = 5; iTRUCK19.18 −Dox: N = 6; iTRUCK19.18 +Dox: N = 5). *p < 0.05, **p < 0.01, ****p < 0.0001 (one-tailed paired t test for B; log rank test for F; one-tailed unpaired t test for D, G, I, J, and L).

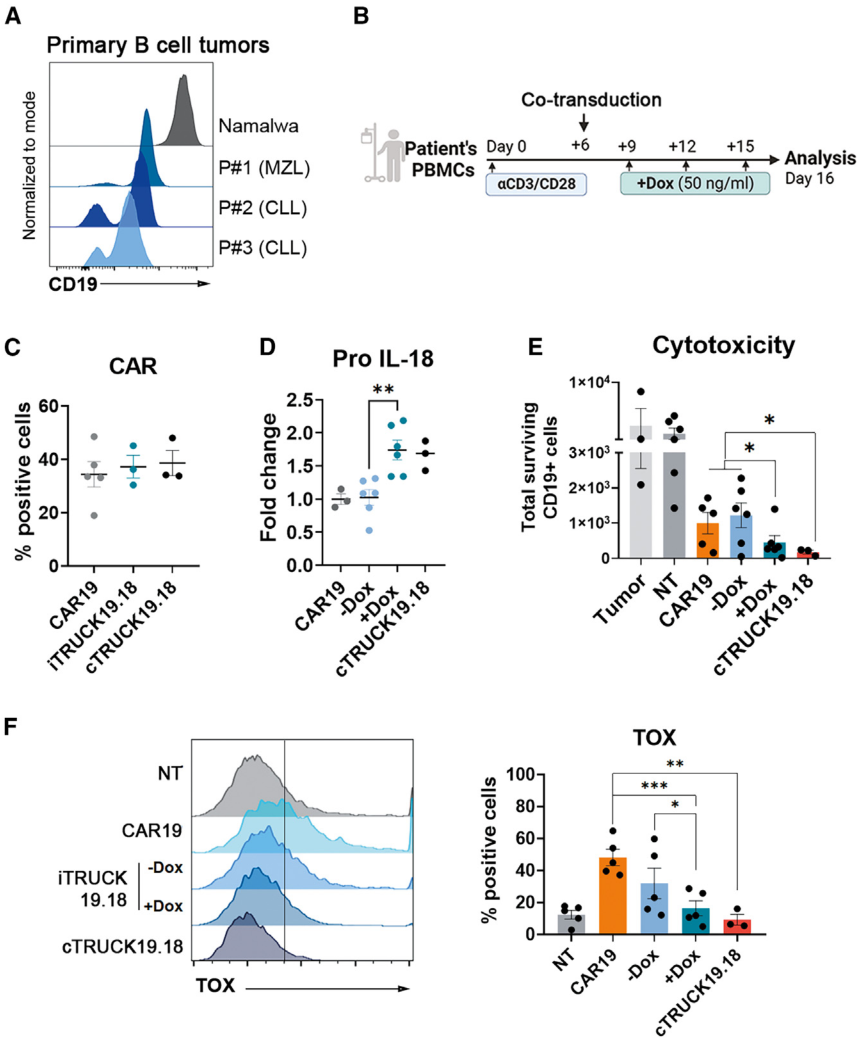

Figure 4. Characterization and lytic capacity of patient-derived iTRUCK19.18 cells against primary B tumors

(A) Representative histograms of CD19 expression of primary tumor sample-derived MZL (patient 1, leukemic mantle cell lymphoma), CLL (patients 2 and 3, chronic lymphocytic leukemia), and Namalwa cells (from Burkitt's lymphoma). (B) Scheme of the generation and analysis of patient-derived CAR-T cells. (C) Percentage of CAR+ cells of patient-derived CAR19, iTRUCK19.18, and cTRUCK19.18 cells (n = 3). (D) Fold change of pro-IL-18 expression of patient-derived CAR19 and iTRUCK19.18 in the absence (−Dox) or presence (+Dox) of 50 ng/mL Dox and cTRUCK19.18 (n = 3). (E) Surviving CD19+ tumor cells following encounter with NT, patient-derived iTRUCK19.18 in the absence (−Dox) or presence (+Dox) of 50 ng/mL Dox and cTRUCK19.18 cells, at an E:T ratio of 1:5 and measured after 13 h of co-culture. (F) Representative histograms (left) and graph (right) showing the percentage of Tox expression of patient-derived NT, CAR19, or iTRUCK19.18 cells without and with Dox and cTRUCK19.18 cells. Analysis performed on total CD3+ T cells (NT, iTRUCK19.18 −Dox, and +Dox: n = 5; CAR19: n = 5; cTRUCK19.18 cells: n = 3). $^{*}p < 0.05$, $^{**}p < 0.01$ (two-tailed paired t test).

applications of LOP18 LVs to enhance CAR-T therapy against solid tumors. In this line, we use iTRUCK19.18 against a metastatic PDAC model engineered to express CD19 (MIA-PaCa2 cells 70% CD19) previously developed by our group.[40] We analyzed the anti-tumor efficacy of iTRUCK19.18 cells *in vitro* after serial tumoral challenges (Figure 5A). The results revealed an increase in the antitumoral potency of iTRUCK19.18 cells with Dox compared with those without Dox in every encounter analyzed (Figures 5B, S6A, and S6B). This enhanced effectiveness was linked to the maintenance of a less-differentiated phenotype ($T_{naive/SCM}$+T_{CM}) observed in the IL-18-producing cells starting from the second encounter (Figures 5C and S6C). In addition, we found no differences in PD1 or TIM3 exhaustion markers regardless of the Dox addition (Figures 5D and S6D), so we could also confirm that IL-18-releasing iTRUCK19.18 cells not only do not accelerate T cell exhaustion but also retain T cells in a memory phenotype that increases their antitumoral potency. The role of IL-18 enhancing the potency of CD19 CAR-T cells was further confirmed by demonstrating similar or even higher effects using cTRUCKs19-18, which constitutively express IL-18 (Figure S6B). Finally, we generated a murine model where we orthotopically implanted MIA-PaCa CD19+ GFP-Nluc cells into the pancreas of NSG mice. Upon tumor development (7 days later), mice were treated with 2 × 10^6 CD19 CAR-T cells or iTRUCK19.18 cells in the presence or absence of Dox (administered orally). Although all treated mice reduced tumor progression (Figures 5E and 5F), those inoculated with iTRUCK19.18 cells and administered with Dox were the only ones where no tumor cells were detected in the pancreas (Figure 5G).

These findings collectively support the therapeutic potential of the LOP system in CAR-T cells for treating different tumor contexts by exogenously controlling IL-18 through Dox administration.

IL-18 induction allows the control of the polarization of pro-tumoral macrophages toward an antitumoral phenotype

As mentioned before, the TME constitutes a major barrier to the clinical efficacy of CAR-T therapy not only against solid tumors, but also against hematological malignancies, resulting in suboptimal outcomes. Within the TME, tumor-associated macrophages play a pivotal role, characterized by an M2 phenotype that exerts potent immunosuppressive effects, thereby constraining the functionality of CAR-T cells.

It has been demonstrated that the expression of IL-18 by CAR-T cells reduced the quantity of M2 macrophages in murine models.[26]

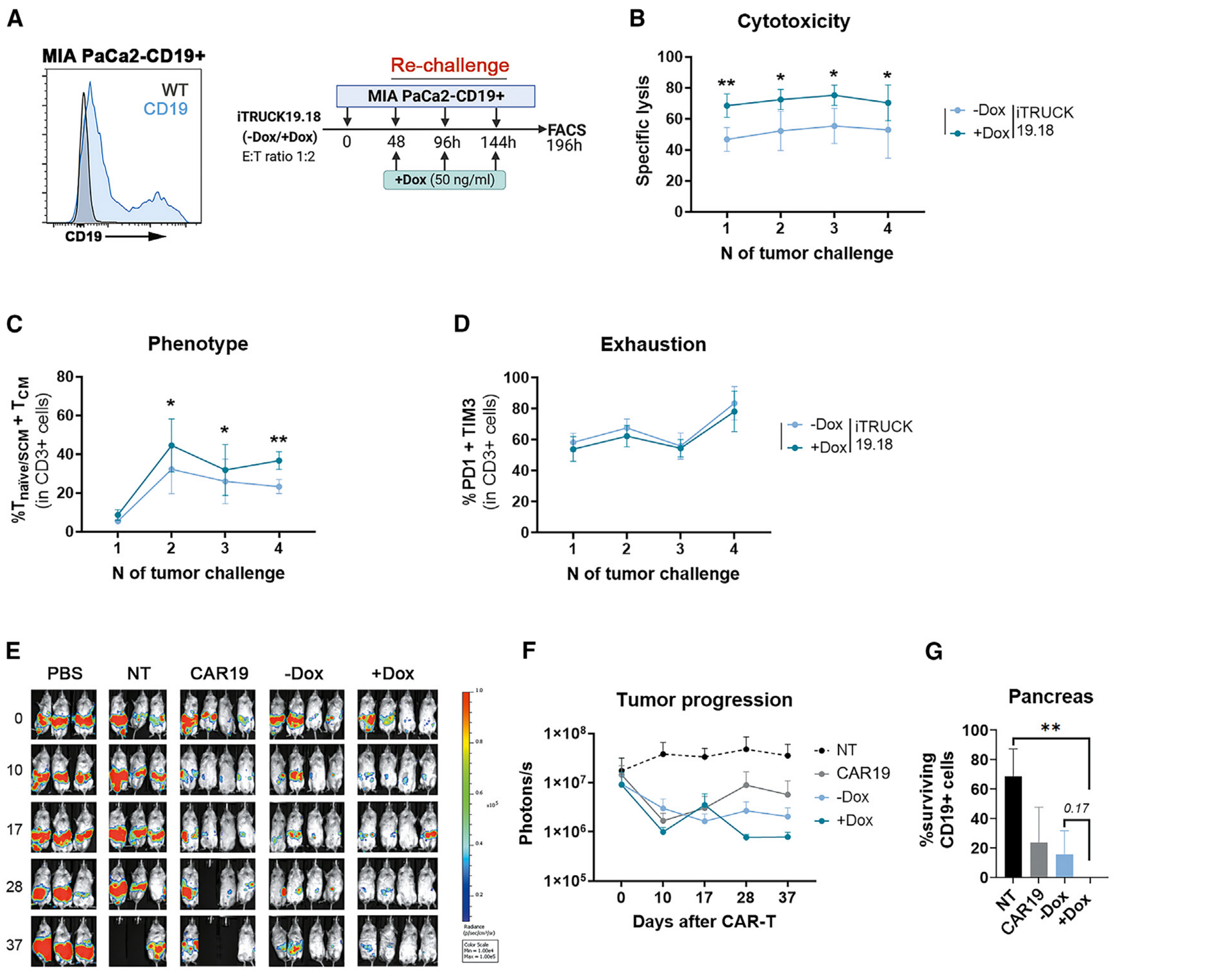

Figure 5. *In vitro* and *in vivo* efficacy of iTRUCK19.18 cells against CD19+ pancreatic ductal adenocarcinoma tumor model

(A) Experimental procedure of the *in vitro* cytotoxicity assay with an artificial model of PDAC cells, MIA-PaCa2-CD19+. Left: histogram showing CD19 expression in MIA-PaCa2 WT (gray) or CD19+ (blue), as target of CAR19 and iTRUCK 19.18. Right: iTRUCK19.18 cells in the absence or presence of Dox (50 ng/mL) were co-cultured at an effector:target ratio of 1:2 with MIA-PaCa2-CD19+ cells. Tumor re-challenges and FACS analysis were performed every 48 h. (B) Specific lysis over four tumor encounters of iTRUCK19.18 −Dox (light blue) and +Dox (dark blue) compared with NT (n = 5). (C) Proportion of $T_{naive/SCM}$ and T_{CM} from −Dox (light blue) and +Dox (dark blue) iTRUCK19.18 after 48 h of every tumor encounter (n = 5). Analysis performed on total CD3+ T cells. (D) Proportion of PD1+ TIM3+ cells inside the CD3+ population, analyzed at every tumor encounter (n = 5). (E) Bioluminescence of tumor progression *in vivo* up to day +37. PBS (N = 3), NT (N = 3), CAR19 (N = 4), iTRUCK19.18 (−Dox, N = 4), and iTRUCK19.18 (+Dox, N = 4). Dox was added at the moment of the inoculation. (F) Tumor progression (photons/s) in mice treated with NT, CAR19, or iTRUCK19.18 without and with Dox. (G) Percentage of tumor cells in the pancreas of mice treated with NT, CAR19, or iTRUCK19.18 without and with Dox. $^{*}p < 0.05$, $^{**}p < 0.01$ (two-tailed paired t test for B and C, and one-tailed unpaired t test for G).

Consequently, we sought to investigate whether controlling the expression of IL-18 would also enable us to regulate the polarization of human M2 pro-tumoral macrophages toward M1 antitumoral macrophages (Figure S7A and following the gating strategy described in Figure S7B). We therefore generated iTRUCK19.18 cells and cTRUCK19.18 and enriched the monocytes from the same donor, which were then differentiated into an M2 phenotype (Figure 6A). Following co-culture of iTRUCK19.18 or cTRUCK19.18 with M2 macrophages (CD206+CD11c−) with M2 macrophages and MIA-PACA-CD19+, we observed the polarization of the macrophages toward M1 (CD206−CD11c+) antitumoral phenotype only in the presence of Dox (Figure 6B). Interestingly, the incubation of M2 macrophages with cTRUCK19.18 expressing IL-18 constitutively had a similar effect (although higher) as compared with iTRUCK19.18 in the presence of Dox (Figure S7C).

Furthermore, we evaluated how the release of IL-18 affected the state of iTRUCK19.18 cells when co-cultured with macrophages. Interestingly, we observed again that iTRUCK19.18 cells exposed to Dox displayed a less differentiated phenotype, with a higher proportion of $T_{naive/SCM}$ and T_{CM} cells, in contrast to cells without Dox (Figure 6C). No significant differences were found in the expression of PD1 and

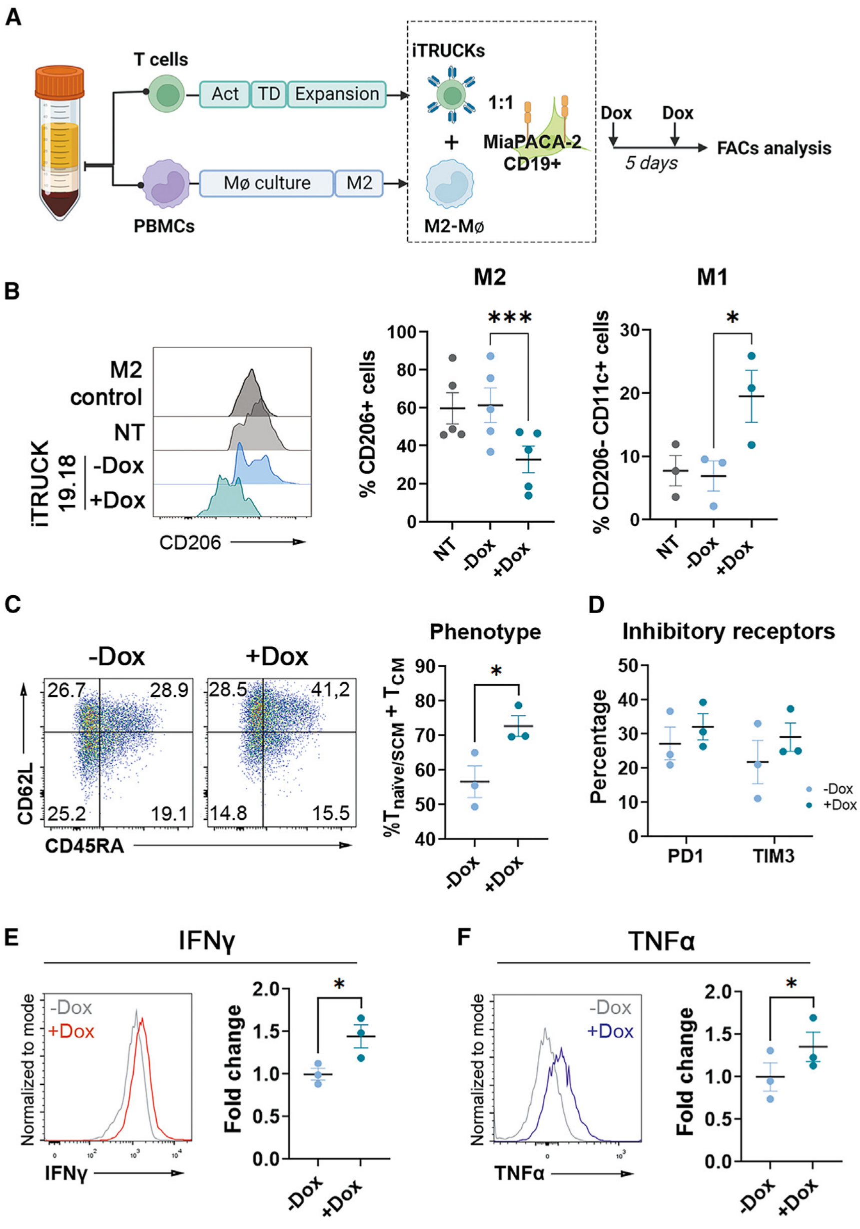

Figure 6. Dox addition to iTRUCK19.18 cells induces polarization of primary human M2 macrophages toward an M1 phenotype

(A) Experimental diagram of the generation of iTRUCK19.18 and M2-polarized macrophages from the same donor. When indicated, Dox (50 ng/mL) was added at the beginning of the co-culture. (B) Left: representative histograms corresponding to CD206 expression of macrophages co-cultured with the different groups of T cells. Center and right: percentage of CD206+ M2 (center) and M1 (right) macrophages when co-cultured with NT cells, iTRUCK19.18 cells without and with Dox, and with MIA-PaCa2 CD19+ cells at ratio 1:1 MIA-PaCa2 vs. CAR-T (M2: *n* = 5; M1: *n* = 3). (C) Representative dot plots and quantification of the proportion of $T_{naive/SCM}$ and T_{CM} cells of iTRUCK19.18 cells in the presence of macrophages with and without Dox after 5 days of the co-culture (*n* = 3). Analysis performed on total CD3+ T cells. (D) Percentage of PD1 and TIM3+ cells inside the CD3+ population of iTRUCK19.18 cells after the co-culture in the presence (dark blue) or absence (light blue) of Dox (*n* = 3). Representative histograms and fold change of (E) IFN-γ and (F) TNF-α expression (compared with those without Dox) of iTRUCK19.18 cells in co-culture with macrophages with and without Dox (*n* = 3). $^{*}p < 0.05$, $^{***}p < 0.001$ (two-tailed paired t test for B; one-tailed paired t test for C, D, E, and F).

TIM3 (Figure 6D). Of note, we validated that the induction of IL-18 in this microenvironment led to a Dox-dependent increase in the production of IFN-γ (Figure 6E) and TNF-α (Figure 6F) by iTRUCK19.18 cells.

All together these data indicate that iTRUCK19.18 cells can be externally controlled not only to enhance their potency but also to act on other immune cells, such as macrophages, to potentially re-shape the TME.

DISCUSSION

Clinical experience has highlighted that CAR-T therapy still has significant room for improvement when applied to patients with aggressive/resistant neoplasms. Conventional CAR-T cells have not demonstrated sufficient efficacy in many cancers, leading to frequent relapses in liquid neoplasms and an almost complete lack of therapeutic effect in solid tumors.

One of the most promising approaches to enhance the effectiveness of CAR-T therapy in recurrent neoplasms is the development of fourth generation CAR-T cells (TRUCKs). In clinical trials, these TRUCKs have shown promising results by overexpressing cytokines such as IL-12 (NCT02498912), IL-15 (NCT03579888), and IL-18 (NCT04684563), which exert a strong immune-modulating action and allow for the restructuring of the TME. TME plays a pivotal role in causing premature dysfunction of CAR-T cells. Consequently, strategies aimed at mitigating its immunosuppressive actions are emerging as interesting alternatives to improve treatment outcomes.

Despite the promising therapeutic effect of TRUCKs that overexpress cytokines in hard-to-treat tumors, their continuous immunomodulatory action can also lead to serious toxicities. Sustained administration of IL-12 and IL-15 has been linked to severe toxicity events,[41,42] while the constitutive expression of IL-18 has been associated not only with specific toxicity events[31,43] but also with the onset of autoimmune disorders[32] and IFN-γ-independent toxicities.[34] Although very elegant mechanisms have been proposed to increase the potency

of CAR-T cells by generating autocrine loops through the expression of membrane-anchored IL-18[44] or by expressing a GM-CSF/IL-18 chimeric receptor,[45] IL-18 secretion also allows for a paracrine effect in the modification of immune populations, allowing the modification over the TME and tumor sensitization by: (1) increasing NK cell-mediated immunity,[46] (2) reducing immunosuppressive macrophages, Treg cells, and immunosuppressive dendritic cells,[26] and (3) increasing bystander activation of T cells[29] (something that does not occur with the two previous strategies); although, as mentioned, the permanent secretion of the cytokine may result in systemic toxicity.[43] Contrasting with the potential safety concerns related with IL-18 secretion, no serious side effects have been observed in the NCT04684563 clinical trial (using TRUCKs expressing IL-18 constitutively).[30] These are very promising results, but they should be approached with caution. Monitoring IL-18 secretion toxicities over longer periods and in larger populations are critical. In addition, it is important to recognize that these toxicities may vary based on factors such as CAR-T cell design, cancer type, and patient status. Therefore, it is essential to control the expression of these cytokines in CAR-T cells to achieve an appropriate balance between efficacy and safety.

The primary mechanism for controlling these cytokines is the use of activation-inducible promoters, particularly the NFAT promoter, which has been applied to regulate IL-12 or IL-18 (NCT03542799)[26] among others. While yielding promising results, there have been reported cases where the NFAT promoter has failed to effectively regulate IL-12 expression, resulting in *in vivo* toxicity.[10,11] This is because this promoter is activated in response to T cell activation, which may not be exclusively dependent on the antigenic recognition by the CAR.

As an alternative, approaches using Tet-On systems, which enable inducible gene expression through Dox, have shown promising results, although their use has been relatively underexplored. Traditional Tet-On systems present certain significant limitations. First, the most commonly used Dox induction systems (Tet-On-3G system by Takara Bio, or the system proposed by Alsaieedi and colleagues) require very high concentrations of Dox (in this latter case, 2 mg/mL) for *in vivo* induction.[11] This could potentially contribute to the development of long-term bacterial resistance due to prolonged or intermittent exposure.[15,47] In addition, most of these systems require transactivator proteins. These bacterial/viral chimeras are necessary to trigger transgene transcription. This point is crucial because important safety considerations must be made since these elements appear to hijack transcription factors in a non-expected manner. When combined with their ability to bind to *pseudo*-tetO sites throughout the genome, they can cause undesired and nonspecific transactivation of genes.[16–18,48] In addition, Schmitt et al. have described alterations due to transactivators in activated, memory, and regulatory splenic T cells subsets in transgenic mice carrying a Tet-On transactivator after only 6 days in the presence of Dox.[49] Altogether, the use of transactivators seems to strongly hinder safe clinical applications. To prevent these potential toxicities for safer ATPMs, we used the LOP system to generate αCD19 CAR-T cells able to induce IL-18 expression under Dox. This system, which not only relies on transactivator proteins, allows for a closer approach to clinical application. Moreover, the system allows *in vivo* induction under ultra-low doses of Dox at nanogram level.[23] To our knowledge, we present the first CAR-T cells able to exogenously induce IL-18 expression, and the first Dox-inducible/transactivator-free TRUCK (iTRUCK19.18). As a downside, contrary to rtTA systems, LOP requires 6–9 days to achieve complete regulation due to the mechanism of action that requires the TetR to accumulate at enough concentrations to block the CMVTetO promoter. Although for some approaches this delay can be a problem, IL-18 secretion during CAR-T cell activity is not a problem since the product characteristics are not affected by IL-18 presence, even showing an improved phenotype (Figure 1F).

iTRUCK19.18 cell generation through co-transduction with CAR19 and LOP18 LVs leads to heterogeneous cell populations. Although obtaining more homogeneous populations would be ideal,[50] in the case of IL-18, its importance is diminished since CAR+IL-18− cells exhibit antitumor activity per se, while CAR-IL-18+ cells provide support to all T cells, whether they carry the CAR or not, as all T cells express IL-18 receptors. This bystander effect enhances their antitumor activity, as previously demonstrated by Hu and colleagues.[28] Moreover, using two independent vectors provides versatility to the system, enabling its application in combination with any synthetic CAR, patient-derived TCR, or even on TILs without the need to modify the lentiviral backbone. It is important to note that, despite the absence of clear evidence for a superior method of expressing multiple transgenes on T cells, co-transduction has been demonstrated as safe,[51] and has produced solid results, even better than using bicistronic vectors in some cases,[52,53] recently reaching the clinical stage (NCT02443831).[54] As a basis for this clinical trial, Kokalaki et al. demonstrated that co-transduction to express multiple transgenes in T cells reduced the risk of loss of stability in the expression of each transgene, and they showed that the heterogeneity between independent batches was minimal.[55] In the same direction, we observed that the production of iTRUCK19.18 cells through co-transduction does not significantly affect parameters related to activation and phenotype compared with the production of conventional CAR-T cells. However, GMP production of TRUCKs mediated by co-transduction presents some limitations, primarily based on the cost associated with producing two batches of LVs under GMP conditions. This limitation can be mitigated by using DNA transposon-based delivery systems (based on predominant technologies such as Sleeping Beauty or PiggyBac). Even with this in mind, it is necessary to emphasize that generating TRUCKs products through co-transduction is compatible with major semi- and fully automated GMP production systems, such as GRex, Xuri, or CliniMACS Prodigy, without significantly altering the manufacturing protocol. In short, co-transduction can overcome some technical difficulties of using bicistronic vectors that have led to poor clinical data, and allows lower costs compared with two full-cell product manufacturers for pooled co-infusion.

It is interesting to note that the secretion of IL-18 by iTRUCK19.18 cells is detected when both cells are activated and Dox is added, following the natural process of IL-18 processing and secretion

observed in other cell types.[39] Interestingly, in the absence of activation but in the presence of Dox, intracellular pro-IL-18 was detected, and functional effects on T cells were observed. These results suggest that, even without activation, iTRUCK19.18 cells can express low levels of IL-18 upon Dox addition, inducing certain functional effects. Importantly, these findings strongly indicate that the IL-18 produced by iTRUCK19.18 cells is bioactive, and that the combination of the double safety mechanism activation/Dox allows for the generation of a safer system compared with the standardized use of NFAT-based promoters. Our data also revealed that the intracellular levels of GzB and TNF-α increase more in unstimulated iTRUCKs19-18 cells than in stimulated cells after the addition of Dox. This unexpected result may be linked to the presence of membrane-bound IL-18 in unstimulated cells as observed in macrophages under specific conditions.[56] This could explain the induction of proinflammatory cytokines after the addition of Dox in unstimulated iTRUCKs19-18 cells despite the absence of secreted IL-18. These data open the door to potential membrane-bound IL-18 in transgenic T cells expressing IL-18 that must be analyzed in detail.

To target different aggressive tumor contexts, we employed iTRUCK19.18 cells in both liquid and CD19-engineered solid tumor models, as well as in patient tumor samples. Analysis in a lymphoma model (Namalwa) demonstrated that iTRUCK19.18 cells exhibit superior antitumoral activity compared with conventional CAR-T cells (in line with the observations of Hu et al. and Avanzi et al. targeting CD19+ neoplasms),[28,29] and this activity can be controlled using Dox. However, we must consider that IL-18 will also increase TCR-mediated antitumoral effects, and this is a limitation of the study, since we are measuring total antitumoral activity upon the addition of Dox in a mixed population.

After confirming the effectiveness of our system in an aggressive hematological neoplasm model, we assessed its activity in a solid tumor model to verify that the effectiveness is not dependent on the tumor type. Once again, we observed a significant increase in antitumoral potency using a CD19+ pancreatic tumor model (in line with studies reporting increased antitumoral activity of IL-18-releasing CAR-T cells against solid tumors).[26,57] Consistent with the lymphoma model, we found a less differentiated phenotype, leading to heightened antitumoral efficacy. Apart from the augmented activation, we hypothesized that this improved phenotype and retained cellular exhaustion contribute to enhanced T cell fitness, ultimately resulting in superior antitumoral potency, aligning with recent findings reported by Jaspers et al.[57] Additional studies are warranted to elucidate the inherent mechanistic factors linked to the exogenous expression of IL-18 on T cells. This phenomenon likely correlates with a decrease in markers indicative of terminal differentiation such as Tox (Figure 4F).

The generation of iTRUCK19.18 cells from patient T cells confirmed the feasibility of co-transduction to achieve sufficient expression levels of both transgenes without any significant alteration on T cell fitness and provides a clinically closer approach of the IL-18-derived increased antitumor potency of iTRUCK19.18.

The demonstration that iTRUCK19.18 can also be used to eliminate pancreatic tumor cells expressing CD19 both *in vitro* and *in vivo* opens the doors to use LOP18 to improve potency not only of other CAR-T cells directed to different tumor antigens (HER2, CEA, BCMA, etc.), but also other immunotherapies such as TILs.

Our findings clearly showed the positive effects of Dox-induced IL-18 on T cells, resulting in an increased antitumoral activity of iTRUCK19.18 cells. However, IL-18 has a plethora of biological actions on different cells that are crucial over the TME. We therefore used an *in vitro* model for the same donor to demonstrate that protumoral human M2 tumor-associated macrophages can be polarized to an antitumoral M1 phenotype by iTRUCK19.18 cells in a Dox-dependent manner. This is consistent with what was observed by Chmielewski and colleagues in a murine model.[26] Overall, this suggests that IL-18 may potentially reshape the TME, enhancing the immune-activating and cytotoxic function of CAR-T cells.

Our results indicate that controlling the release of IL-18 by CAR-T cells also allows for controlling their antitumor potency in different tumor contexts. Cytokine control through an ultra-low dose Dox-inducible system free of transactivators (LOP) allows for the generation of a safe and more effective TRUCKs product, representing an alternative to conventional CAR-T cells for treating patients with aggressive type B neoplasms that require increased potency without compromising safety.

MATERIALS AND METHODS

Cell lines

HEK293T (human embryonic kidney-derived cells, ATCC CRL-11268) and MIA-PaCa2 (human pancreatic adenocarcinoma cells, ATCC CRL-1420) cell lines were cultured with DMEM (Dulbecco's modified Eagle's medium) (Biowest) supplemented with 10% fetal bovine serum (FBS) (Gibco) and 1% penicillin/streptomycin (P/S) (Gibco). Jurkat (acute T cell leukemia, ATCC TIB-152) and Namalwa (Burkitt's lymphoma cells, ATCC CRL-1432) cell lines were grown in RPMI-1640 (Roswell Park Memorial Institute) (Biowest) supplemented with 10% FBS and 1% P/S. Cell lines were routinely tested for mycoplasma.

Isolation and culture of primary T cells

Peripheral blood samples from healthy donors and patients were provided by the Hematology and Hemotherapy Unit of the Reina Sofía University Hospital (Córdoba, Spain) and Virgen de las Nieves University Hospital (Granada, Spain) under informed consent, following the guidelines of the ethics committee and in accordance with Spanish regulations (RD-L 9/2014). T cells from healthy donors were obtained from PBMCs. Blood was diluted 1/2–1/4 in PBS (Biowest) and PBMCs were isolated using Ficoll gradient centrifugation (Cytiva) at $400 \times g$ for 20 min without brake or acceleration. The mononuclear lymphocyte layer was collected and washed with PBS. Cells were cultured at 2×10^6 cells/mL in TexMACS medium (Miltenyi Biotec) supplemented with 10 ng/mL of IL-7 and IL-15 (Miltenyi Biotec) and 1% P/S (Biowest) in at 37°C and 5% CO_2.

Patient PBMCs were obtained from 5 to 8 mL of blood from patients diagnosed with MZL or CLL before treatment. PBMCs were isolated as described before and cultured at 2×10^6 cells/mL with TexMACS medium supplemented with 1% P/S, 5% human AB serum (Biowest), and 40 IU/mL of IL-2 (Miltenyi Biotec). Twenty-four hours later, TransAct (1:100) was added for 6 days prior to transduction. Expansion after transduction was performed with TexMACS supplemented with 10 ng/mL of IL-7 and IL-15, 1% P/S, and 5% human AB serum. Patient B cells were maintained in TexMACS supplemented with 5% human AB serum for cytotoxicity assays.

LVs

EF1α-A3B1-19BBz (CAR19, ARI-0001) plasmid was kindly provided by Dr. Manel Juan and Dr. Maria Castella from Hospital Clínic (Barcelona, Spain). CIL18ETIS2 (LOP18) plasmid was generated by designing and incorporating hIL-18 sequence (RefSeq Transcript ID GenBank: NM_001386420.1, synthesized by ATG:biosynthetics) flanked by AscI/SbfI sites into the CELETIS2[23] plasmid, replacing the eGFP-2A-Nluc region. EF1α-IL18 LV was generated by cloning the hIL-18 under the EF1α promoter with tEcoRI and SbfI sites in a pUC19 from ATG:biosynthetics.

LVs production and titration

HEK293T cells were co-transfected with the transfer plasmid, plasmid pCMVDR8.91, and plasmid pMD.G as described previously[22] using polyethylenimine (Alfa Aesar). Viral supernatants were collected 48 and 72 h post transfection and concentrated 100× by ultracentrifugation (90,000 × g, 4°C, 2 h) and functional titer was determined in Jurkat cells as described.[58]

Generation of iTRUCK19.18

Primary T cells were activated with T cell TransAct (Miltenyi Biotec) and 48 h later co-transduced by a mixture of CAR19 LVs and LOP18 LVs as described previously.[38] In brief, LVs were mixed and cells were added for spinoculation (800 × g, 32°C, 1 h). Five hours later, cells were washed and plated at a density of 1×10^6 with TexMACS medium (Miltenyi Biotech) supplemented with 10 ng/mL of IL-7 and IL-15 (Miltenyi Biotech). CAR and pro-IL-18 expression levels in iTRUCK19.18 were characterized by FACS, and functional studies were performed with a bulk population expressing similar levels of both transgenes.

Flow cytometry

CAR expression was analyzed using a primary goat IgG1 antibody that binds to the murine Fab conjugated with biotin (1:100, Jackson Immunoresearch, 115-065-072) and Streptavidin-APC (1:330, eBioscience, 17-4317-82). In brief, an anti-Fab antibody was added for 40 min. After washing, Streptavidin-APC was added for 30 min. After 15 min in the presence of Streptavidin, extracellular antibodies were added.

For the immunophenotyping of primary T cells, the following monoclonal antibodies were used: CD45RA-FITC (HI-100, 1:200), CD62L-PE-Cy7 (DREG56, 1:200), CD3-PerCP-Cy5.5 (OKT3, 1:200), CD4-eFluor450 (RPA-T4, 1:100), PD1-APC (MIH4, 1:50), LAG-3-PE (3DS223H, 1:100), and TIM-3-eFluor780 (F38-2E2, 1:50), all from eBioscience (Thermo Fisher Scientific). All these analyses were performed gating on CD3+. For measuring AICD markers, the following monoclonal antibodies were used: anti-human CD253 (TRAIL)-PE (1:100, BD Biosciences, RIK-2), anti-Hu CD95 (APO-1/Fas)-APC (1:200, eBiosciences, DX2), Annexin V/7ADD (eBiosciences), and Fas Ligand Ms Anti-Hu mAb-FITC (1:200, Life Technologies, SB93a). For the characterization of primary macrophages, CD206-FITC (19.2, 1:100), CD11c-PE (3.9, 1:50), and CD14-PerCP-Cy5.5 (61D3, 1:200) from eBiosciences (Thermo Fisher Scientific) were used after blocking FcR receptors with FcR Blocking during 15 min on ice (1:100, Miltenyi Biotec).

Intracellular staining was performed using the Fix & Perm kit (Nordic MUbio), following the manufacturer's recommendations. Anti-hIL-18 Propeptide-PE (1:20, R&D Systems, 74801), pCD3ζ-PE (1:100, eBioscience, Tyr142, 3ZBR4S), anti-Hu IFNgamma-FITC (1:100, eBioscience, 4S.B3), anti-Hu Granzyme B-eFluor 450 (1:100, eBioscience, N4TL33), anti-Hu IL-2-PE-Cyanine7 (1:100, eBioscience, MQ1-17H12), anti-Hu TNFalpha-APC (1:100, eBioscience, Mab11), anti-Hu Perforin-FITC (1:100, eBiosciences, d69), and anti-Tox-PE (REA473, 1:100) were used for different assays.

Mice samples were obtained by mechanical disruption (bone marrow, spleen, and liver) or after a Percoll (GE Healthcare) gradient (brain). Fcγ receptors were blocked using murine αCD16/CD32 (Thermo Fisher Scientific), human FcR Blocking (Miltenyi Biotec), and 5% mouse serum (Sigma-Aldrich). All stainings were performed in darkness, on ice, and washes were performed with FACS buffer (PBS + 3% BSA + 2 mM EDTA) at 400 × g for 5 min, unless otherwise indicated. Cytometers used for acquisition were FACSCanto II and FACSVerse (BD Biosciences), performing exclusion by singlets, as well as live/dead cells using 4′,6-diamidino-2-phenylindole (Thermo Fisher Scientific). In the case of intracellular stainings, cell viability was determined using Ghost Dye Violet 510 (Tonbo Biosciences). Absolute cell quantification was performed using CountBright Absolute Counting Beads (Thermo Fisher Scientific). Data analysis was performed using FlowJo v.10 software (TreeStar).

IL-18 secretion assay

For the study of secreted hIL-18, we used HEKBlue IL-18 cells (Invitrogen) that allow the detection of bioactive hIL-18 (10 pg/mL to 1 ng/mL). In brief, 3×10^5 T cells were plated per condition in 150 μL of TexMACS medium (Miltenyi Biotec) without cytokines. Cells were activated with TransAct (1:150, Miltenyi Biotec) or PBS (negative activation control) for 24 h. Supernatants were collected and frozen and then analyzed following the manufacturer's instructions.

Polarization assay

PBMCs from healthy donors were thawed in a 96-well plate at a density of 2×10^6 cells/mL. After 24 h, suspended cells (mostly T cells) were separated and cultured with TexMACS (Miltenyi Biotec) supplemented with IL-7 and IL-15 (Miltenyi Biotec), while the adherent

cells (monocytes/macrophages) were cultured with RPMI-1640 supplemented with 10% FBS (Biowest). Macrophages were supplemented with either (1) 50 ng/mL GM-CSF (PeproTech) (for polarization to an M1 phenotype) or (2) 50 ng/mL M-CSF (PeproTech) (for polarization to an M2 phenotype). Ninety-six hours later, macrophages were supplemented with either (1) 10 ng/mL of *E. coli* lipopolysaccharide (Sigma-Aldrich) for final polarization to M1 or (2) 20 ng/mL of IL-4 (PeproTech) for final polarization to M2. Flow cytometry analysis was performed after 6 days to confirm macrophage polarization.

Cytotoxicity assays

Namalwa cells

Cytotoxicity was performed as described previously.[59] In brief, 7.5×10^4 Namalwa GFP-Nluc cells were co-cultured with T cells in duplicate in 96-well plates (Thermo Fisher Scientific), maintaining an effector-to-target (E:T) ratio of 1:10 (calculated based on CAR+ cells), in TexMACS medium without supplementation. Tumor re-stimulations were performed by adding the same number of tumor cells as initially present.

Primary tumors

Primary CD19+ tumor cells (5×10^4) isolated from peripheral blood of untreated patients with MZL and CLL were co-cultured with T cells from healthy donors or from the same patient, transduced with CAR and LOP18, at three different E:T ratios: 1:1, 1:2, and 1:5 (calculated based on the percentage of CAR+ cells), in duplicate in 96-well plates. Cytotoxicity reading was performed 13 h after co-culturing using flow cytometry. Subsequent re-challenges were performed with 5×10^4 primary CD19+ tumor cells every 24 h up to four re-challenges.

MIA-PaCa2 CD19+ cells

MIA-PaCa2 target cells (7.5×10^3) expressing 100% GFP-Nluc and 70% CD19 were seeded in duplicate in 96-well plates (Thermo Fisher Scientific) the day before adding T cells. Cells were cultured in complete DMEM (Biowest) (+10% FBS, +1% P/S) (Biowest). Next day, target cells were incubated with T cells at an E:T ratio of 1:2 (calculated based on CAR+ cells) in non-supplemented TexMACS medium for 48 h. Tumor re-stimulations were performed by adding the same number of tumor cells as initially present.

Specific lysis was calculated using the following formula: specific lysis = 1 − (%CD19+ cells in CAR condition/%CD19+ in NT condition) × 100; formula adapted from Larson et al.[60]

Cytokine quantification

Cytokine quantification was performed using the MACSPlex Cytokine 12 Kit (Miltenyi Biotec) following the manufacturer's instructions. Log2 (score) was calculated as follows: Log2(secreted cytokine in iTRUCK condition/secreted cytokine in CAR condition), using the mean between donors.

Animal models

All mice were handled according to EU European (2010/63/UE) and local animal wellness regulations (RD1386/2018, RD53/2013), previous revision and approval by the local Ethics Committee. Burkitt's lymphoma murine model was generated as described previously.[61] In brief, 0.3×10^6 Namalwa GFP-Nluc cells were intravenously inoculated into 10- to 12-week-old NSG mice. Three or 6 days later, CAR-T cells were intravenously injected. In some cases, tumor re-inoculations were performed.

To generate the orthotopic pancreatic tumor model, 8- to 10-week-old NSG mice were injected with MIA-PaCa2 GFP-Nluc cells (70% CD19+) embedded in Matrigel (Corning) into the tail of the pancreas at day −7. On day 0, 2×10^6 CAR-T cells were intravenously injected and tumoral progression was monitored up to 37 days. Mice were euthanized by cervical dislocation when they exhibited a high bioluminescence signal, a weight loss of >20% of their initial weight for Namalwa model, showed an increase of >20% of their weight in less than 5 days for MIA-PaCa2 model, or showed clear signs of pain or xenoGVHD. Dox (1,000 or 500 ng/mL) was provided dissolved in sterile water and changed twice a week. In addition, Dox was provided in strawberry jelly prepared with sterile water.

Bioluminescence analysis

Bioluminescence analysis was performed as described.[23] In brief, furimazine (NanoGlo, Promega) was administered via intraperitoneal (1/150 in PBS) just before acquiring the image using the IVIS Spectrum analyzer (Caliper, PerkinElmer). Images were analyzed using Living Image 3.2 (PerkinElmer) or AURA Imaging 4.0.7 (Spectral Instruments Imaging).

Data analysis

Statistical analyses were performed using GraphPad Prism 9 (Dotmatics). Data are expressed as mean ± SEM. Each n represents an independent donor; each N represents a mouse. Statistical test is indicated in the corresponding figure caption.

DATA AND CODE AVAILABILITY

All data are available in the main text or in the supplemental information. LOP-18 LV is available under a material transfer agreement with LentiStem Biotech.

ACKNOWLEDGMENTS

We acknowledge Dr. Manel Juan and Dr. Maria Castella for kindly providing CAR19 ARI-0001 LV. We also thank Dr. Paulina Rybakowska, Dr. Araceli Aguilar, and Paula Heredia for their support with human samples; Ana Fernández-Ibáñez for her support with the IVIS Spectrum Analyzer, and all supporting Units from GENYO.

The work has been funded by the Instituto de Salud Carlos III (ISCIII) and the European Regional Development Fund (FEDER), grant PI21/00298 (to F.M.); Instituto de Salud Carlos III (ISCIII) – NextGenerationEU funds – actions of the Recovery and Resilience Mechanism, Red TerAv RD21/0017/0004 (to F.M., J.A.M., and F.P.); Ministerio de Ciencia e Innovación (MICIN), Plan de Recuperación, transformación y resilencia, Centro para el Desarrollo Tecnológico Industrial (CDTI) and European Union-Next Generation EU: grants 00123009/SNEO-20191072 (to F.M.), PMPTA22/00060 (to F.M. and J.R.-M.), and DIN2018-010180 (to P.J.-L.); Consejería de Salud y Familias (CSyF) – Junta de Andalucía – FEDER/European Cohesion Fund (FSE) for Andalucía: grants 2016000073332-TRA, CARTPI-0001-201, PECART-0031-2020, and CAR-T 2019 00400200101918 (Red RANTECAR) (to F.M. and J.A.M.); Consejería de Economía, conocimiento, empresa y Universidad, grant A-CTS-235-UGR18 (to F.M.); Ministerio de Ciencia e Innovación (MICIN) – líneas estratégicas: grant PLEC2021-008094 (to F.M. and J.A.M.); Fellowship Garantía Juvenil (PEJ2018-001760-A) (to M.C.-G.); and Chair "Doctors Galera-Requena

in cancer stem cell research" (CMC-CTS963) (to J.A.M.). C.P. was supported by the PFIS fellowship from ISCIII (FI21/00161).

AUTHOR CONTRIBUTIONS

Conceptualization, F.M., M.T.-M., P.J.-L., and N.M.-P.; methodology, P.J.-L., M.T.-M., N.M.-P., F.M., and J.A.M.; investigation, P.J.-L., M.T.-M., N.M.-P., C.B.-J., M.C.-G., K.P., P.M., A.H.-B., C.G.-L., S.A.N.-M., J.R.M.-M., and F.P.; resources, F.M., J.M.-B., P.A.G.-S., C.H., J.R.M.-M., and F.P.; writing – original draft, P.J.-L., M.T.-M., and F.M.; writing – review & editing, F.M., M.T.-M., P.J.-L., and F.J.M.-E.; supervision, M.T.-M. and F.M.; funding acquisition, F.M. and J.A.M.

DECLARATION OF INTERESTS

F.M. and P.M. are inventors of the patents entitled "Lent-on-plus system for conditional expression in human stem cells" (PCT/EP2017/078246) and "Insulator to improve gene transfer vectors" (PCT/EP2014/055027). F.M., M.T.-M., and J.A.M. are partners of LentiStem Biotech. P.J.-L., M.T.-M., and C.B.-J. are contractually associated with LentiStem Biotech, a spin-off company that holds the license of the above-mentioned patents.

SUPPLEMENTAL INFORMATION

Supplemental information can be found online at https://doi.org/10.1016/j.omtn.2024.102308.

REFERENCES

1. Melenhorst, J.J., Chen, G.M., Wang, M., Porter, D.L., Chen, C., Collins, M.A., Gao, P., Bandyopadhyay, S., Sun, H., Zhao, Z., et al. (2022). Decade-long leukaemia remissions with persistence of CD4+ CAR T cells. Nature *602*, 503–509.
2. Gu, T., Zhu, M., Huang, H., and Hu, Y. (2022). Relapse after CAR-T cell therapy in B-cell malignancies: challenges and future approaches. J. Zhejiang Univ. - Sci. B *23*, 793–811.
3. Todorovic, Z., Todorovic, D., Markovic, V., Ladjevac, N., Zdravkovic, N., Djurdjevic, P., Arsenijevic, N., Milovanovic, M., Arsenijevic, A., and Milovanovic, J. (2022). CAR T Cell Therapy for Chronic Lymphocytic Leukemia: Successes and Shortcomings. Curr. Oncol. *29*, 3647–3657.
4. Safarzadeh Kozani, P., Safarzadeh Kozani, P., Ahmadi Najafabadi, M., Yousefi, F., Mirarefin, S.M.J., and Rahbarizadeh, F. (2022). Recent Advances in Solid Tumor CAR-T Cell Therapy: Driving Tumor Cells From Hero to Zero? Front. Immunol. *13*, 795164.
5. Mazinani, M., and Rahbarizadeh, F. (2022). CAR-T cell potency: from structural elements to vector backbone components. Biomark. Res. *10*, 70.
6. Fraietta, J.A., Lacey, S.F., Orlando, E.J., Pruteanu-Malinici, I., Gohil, M., Lundh, S., Boesteanu, A.C., Wang, Y., O'Connor, R.S., Hwang, W.T., et al. (2018). Determinants of response and resistance to CD19 chimeric antigen receptor (CAR) T cell therapy of chronic lymphocytic leukemia. Nat. Med. *24*, 563–571.
7. Li, J., Li, W., Huang, K., Zhang, Y., Kupfer, G., and Zhao, Q. (2018). Chimeric antigen receptor T cell (CAR-T) immunotherapy for solid tumors: lessons learned and strategies for moving forward. J. Hematol. Oncol. *11*, 22.
8. Chmielewski, M., and Abken, H. (2020). TRUCKS, the fourth-generation CAR T cells: Current developments and clinical translation. Adv. Cell Gene Ther. *3*, e84.
9. Chmielewski, M., and Abken, H. (2015). TRUCKs: the fourth generation of CARs. Expet Opin. Biol. Ther. *15*, 1145–1154.
10. Zhang, L., Morgan, R.A., Beane, J.D., Zheng, Z., Dudley, M.E., Kassim, S.H., Nahvi, A.V., Ngo, L.T., Sherry, R.M., Phan, G.Q., et al. (2015). Tumor-infiltrating lymphocytes genetically engineered with an inducible gene encoding interleukin-12 for the immunotherapy of metastatic melanoma. Clin. Cancer Res. *21*, 2278–2288.
11. Alsaieedi, A., Holler, A., Velica, P., Bendle, G., and Stauss, H.J. (2019). Safety and efficacy of Tet-regulated IL-12 expression in cancer-specific T cells. OncoImmunology *8*, 1542917.
12. Wang, D. (2018). The essential role of G protein-coupled receptor (GPCR) signaling in regulating T cell immunity. Immunopharmacol. Immunotoxicol. *40*, 187–192.
13. Condotta, S.A., and Richer, M.J. (2017). The immune battlefield: The impact of inflammatory cytokines on CD8+ T-cell immunity. PLoS Pathog. *13*, e1006618.
14. Agnellini, P., Wolint, P., Rehr, M., Cahenzli, J., Karrer, U., and Oxenius, A. (2007). Impaired NFAT nuclear translocation results in split exhaustion of virus-specific CD8+ T cell functions during chronic viral infection. Proc. Natl. Acad. Sci. USA *104*, 4565–4570.
15. Tristan-Manzano, M., Justicia-Lirio, P., Maldonado-Perez, N., Cortijo-Gutierrez, M., Benabdellah, K., and Martin, F. (2020). Externally-Controlled Systems for Immunotherapy: From Bench to Bedside. Front. Immunol. *11*, 2044.
16. Morimoto, M., and Kopan, R. (2009). rtTA toxicity limits the usefulness of the SP-C-rtTA transgenic mouse. Dev. Biol. *325*, 171–178.
17. Perl, A.K., Zhang, L., and Whitsett, J.A. (2009). Conditional expression of genes in the respiratory epithelium in transgenic mice: cautionary notes and toward building a better mouse trap. Am. J. Respir. Cell Mol. Biol. *40*, 1–3.
18. Whitsett, J.A., and Perl, A.K.T. (2006). Conditional control of gene expression in the respiratory epithelium: A cautionary note. Am. J. Respir. Cell Mol. Biol. *34*, 519–520.
19. Sisson, T.H., Hansen, J.M., Shah, M., Hanson, K.E., Du, M., Ling, T., Simon, R.H., and Christensen, P.J. (2006). Expression of the reverse tetracycline-transactivator gene causes emphysema-like changes in mice. Am. J. Respir. Cell Mol. Biol. *34*, 552–560.
20. Benabdellah, K., Gutierrez-Guerrero, A., Cobo, M., Muñoz, P., and Martín, F. (2014). A chimeric HS4-SAR insulator (IS2) that prevents silencing and enhances expression of lentiviral vectors in pluripotent stem cells. PLoS One *9*, e84268.
21. Benabdellah, K., Cobo, M., Muñoz, P., Toscano, M.G., and Martin, F. (2011). Development of an all-in-one lentiviral vector system based on the original TetR for the easy generation of Tet-ON cell lines. PLoS One *6*, e23734.
22. Benabdellah, K., Muñoz, P., Cobo, M., Gutierrez-Guerrero, A., Sánchez-Hernández, S., Garcia-Perez, A., Anderson, P., Carrillo-Gálvez, A.B., Toscano, M.G., and Martin, F. (2016). Lent-On-Plus Lentiviral vectors for conditional expression in human stem cells. Sci. Rep. *6*, 37289.
23. Tristán-Manzano, M., Maldonado-Pérez, N., Justicia-Lirio, P., Cortijo-Gutierréz, M., Tristán-Ramos, P., Blanco-Benítez, C., Pavlovic, K., Aguilar-González, A., Muñoz, P., Molina-Estevez, F.J., et al. (2023). Lentiviral vectors for inducible, transactivator-free advanced therapy medicinal products: Application to CAR-T cells. Mol. Ther. Nucleic Acids *32*, 322–339.
24. Hoshino, T., Wiltrout, R.H., and Young, H.A. (1999). IL-18 is a potent coinducer of IL-13 in NK and T cells: a new potential role for IL-18 in modulating the immune response. J. Immunol. *162*, 5070–5077.
25. Ohtsuki, T., Micallef, M.J., Kohno, K., Tanimoto, T., Ikeda, M., and Kurimoto, M. (1997). Interleukin 18 enhances Fas ligand expression and induces apoptosis in Fas-expressing human myelomonocytic KG-1 cells. Anticancer Res. *17*, 3253–3258.
26. Chmielewski, M., and Abken, H. (2017). CAR T Cells Releasing IL-18 Convert to T-Bet(high) FoxO1(low) Effectors that Exhibit Augmented Activity against Advanced Solid Tumors. Cell Rep. *21*, 3205–3219.
27. Olivera, I., Bolaños, E., Gonzalez-Gomariz, J., Hervas-Stubbs, S., Mariño, K.V., Luri-Rey, C., Etxeberria, I., Cirella, A., Egea, J., Glez-Vaz, J., et al. (2023). mRNAs encoding IL-12 and a decoy-resistant variant of IL-18 synergize to engineer T cells for efficacious intratumoral adoptive immunotherapy. Cell Rep. Med. *4*, 100978.
28. Hu, B., Ren, J., Luo, Y., Keith, B., Young, R.M., Scholler, J., Zhao, Y., and June, C.H. (2017). Augmentation of Antitumor Immunity by Human and Mouse CAR T Cells Secreting IL-18. Cell Rep. *20*, 3025–3033.
29. Avanzi, M.P., Yeku, O., Li, X., Wijewarnasuriya, D.P., van Leeuwen, D.G., Cheung, K., Park, H., Purdon, T.J., Daniyan, A.F., Spitzer, M.H., and Brentjens, R.J. (2018). Engineered Tumor-Targeted T Cells Mediate Enhanced Anti-Tumor Efficacy Both Directly and through Activation of the Endogenous Immune System. Cell Rep. *23*, 2130–2141.
30. Svoboda, J., Landsburg, D.J., Nasta, S.D., Barta, S.K., Chong, E.A., Lariviere, M.J., Shea, J., Cervini, A., Hexner, E.O., Marshall, A., et al. (2024). Safety and efficacy of armored huCART19-IL18 in patients with relapsed/refractory lymphomas that progressed after anti-CD19 CAR T cells. In ASCO Annual Meeting: Journal of Clinical Oncology Logo.
31. Breman, E., Walravens, A.-S., Gennart, I., Velghe, A., Nguyen, T., Violle, B., Huberty, F., Ramelot, N., Twyffels, L., Gauthy, E., et al. (2021). 107 Armoring NKG2D CAR T cells with IL-18 improves *in vivo* anti-tumor activity. J. ImmunoTher. Cancer *9*, A118.

32. Tsutsumi, N., Yokota, A., Kimura, T., Kato, Z., Fukao, T., Shirakawa, M., Ohnishi, H., and Tochio, H. (2019). An innate interaction between IL-18 and the propeptide that inactivates its precursor form. Sci. Rep. *9*, 6160.
33. Baggio, C., Bindoli, S., Guidea, I., Doria, A., Oliviero, F., and Sfriso, P. (2023). IL-18 in Autoinflammatory Diseases: Focus on Adult Onset Still Disease and Macrophages Activation Syndrome. Int. J. Mol. Sci. *24*, 11125.
34. Nakamura, S., Otani, T., Ijiri, Y., Motoda, R., Kurimoto, M., and Orita, K. (2000). IFN-γ-Dependent and -Independent Mechanisms in Adverse Effects Caused by Concomitant Administration of IL-18 and IL-12. J. Immunol. *164*, 3330–3336.
35. Diorio, C., Shraim, R., Myers, R., Behrens, E.M., Canna, S., Bassiri, H., Aplenc, R., Burudpakdee, C., Chen, F., DiNofia, A.M., et al. (2022). Comprehensive Serum Proteome Profiling of Cytokine Release Syndrome and Immune Effector Cell-Associated Neurotoxicity Syndrome Patients with B-Cell ALL Receiving CAR T19. Clin. Cancer Res. *28*, 3804–3813.
36. Rocco, J.M., Inglefield, J., Yates, B., Lichtenstein, D.A., Wang, Y., Goffin, L., Filipovic, D., Schiffrin, E.J., and Shah, N.N. (2023). Free interleukin-18 is elevated in CD22 CAR T-cell-associated hemophagocytic lymphohistiocytosis-like toxicities. Blood Adv. *7*, 6134–6139.
37. Castella, M., Boronat, A., Martín-Ibáñez, R., Rodríguez, V., Suñé, G., Caballero, M., Marzal, B., Pérez-Amill, L., Martín-Antonio, B., Castaño, J., et al. (2019). Development of a Novel Anti-CD19 Chimeric Antigen Receptor: A Paradigm for an Affordable CAR T Cell Production at Academic Institutions. Mol. Ther. Methods Clin. Dev. *12*, 134–144.
38. Tristan-Manzano, M., Maldonado-Perez, N., Justicia-Lirio, P., Cortijo-Gutierrez, M., Tristan-Ramos, P., Blanco-Benitez, C., Pavlovic, K., Aguilar-Gonzalez, A., Munoz, P., Molina-Estevez, F.J., et al. (2023). Lentiviral vectors for inducible, transactivator-free advanced therapy medicinal products: Application to CAR-T cells. Mol. Ther. Nucleic Acids *32*, 322–339.
39. van de Veerdonk, F.L., Netea, M.G., Dinarello, C.A., and Joosten, L.A.B. (2011). Inflammasome activation and IL-1beta and IL-18 processing during infection. Trends Immunol. *32*, 110–116.
40. Tristán-Manzano, M., Maldonado-Pérez, N., Justicia-Lirio, P., Muñoz, P., Cortijo-Gutiérrez, M., Pavlovic, K., Jiménez-Moreno, R., Nogueras, S., Carmona, M.D., Sánchez-Hernández, S., et al. (2022). Physiological lentiviral vectors for the generation of improved CAR-T cells. Mol. Ther. Oncolytics *25*, 335–349.
41. Leonard, J.P., Sherman, M.L., Fisher, G.L., Buchanan, L.J., Larsen, G., Atkins, M.B., Sosman, J.A., Dutcher, J.P., Vogelzang, N.J., and Ryan, J.L. (1997). Effects of single-dose interleukin-12 exposure on interleukin-12-associated toxicity and interferon-gamma production. Blood *90*, 2541–2548.
42. Miller, J.S., Morishima, C., McNeel, D.G., Patel, M.R., Kohrt, H.E.K., Thompson, J.A., Sondel, P.M., Wakelee, H.A., Disis, M.L., Kaiser, J.C., et al. (2018). A First-in-Human Phase I Study of Subcutaneous Outpatient Recombinant Human IL15 (rhIL15) in Adults with Advanced Solid Tumors. Clin. Cancer Res. *24*, 1525–1535.
43. Ng, B.D., Rajagopalan, A., Kousa, A.I., Fischman, J.S., Chen, S., Massa, A.R., Elias, H.K., Manuele, D., Galiano, M., Lemarquis, A.L., et al. (2024). IL-18-secreting multi-antigen targeting CAR T-cells eliminate antigen-low myeloma in an immunocompetent mouse model. Blood *144*, 171–186.
44. Blokon-Kogan, D., Levi-Mann, M., Malka-Levy, L., Itzhaki, O., Besser, M.J., Shiftan, Y., Szöőr, Á., Vereb, G., Gross, G., Abken, H., and Weinstein-Marom, H. (2022). Membrane anchored IL-18 linked to constitutively active TLR4 and CD40 improves human T cell antitumor capacities for adoptive cell therapy. J. Immunother. Cancer *10*, e001544.
45. Lange, S., Sand, L.G.L., Bell, M., Patil, S.L., Langfitt, D., and Gottschalk, S. (2021). A Chimeric GM-CSF/IL18 Receptor to Sustain CAR T-cell Function. Cancer Discov. *11*, 1661–1671.
46. Senju, H., Kumagai, A., Nakamura, Y., Yamaguchi, H., Nakatomi, K., Fukami, S., Shiraishi, K., Harada, Y., Nakamura, M., Okamura, H., et al. (2018). Effect of IL-18 on the Expansion and Phenotype of Human Natural Killer Cells: Application to Cancer Immunotherapy. Int. J. Biol. Sci. *14*, 331–340.
47. Grossman, T.H. (2016). Tetracycline Antibiotics and Resistance. Cold Spring Harbor Perspect. Med. *6*, a025387.
48. Hackl, H., Rommer, A., Konrad, T.A., Nassimbeni, C., and Wieser, R. (2010). Tetracycline regulator expression alters the transcriptional program of mammalian cells. PLoS One *5*, e13013.
49. Schmitt, A., Schulze-Osthoff, K., and Hailfinger, S. (2018). Correspondence: T cells are compromised in tetracycline transactivator transgenic mice. Cell Death Differ. *25*, 634–636.
50. Cordoba, S., Onuoha, S., Thomas, S., Pignataro, D.S., Hough, R., Ghorashian, S., Vora, A., Bonney, D., Veys, P., Rao, K., et al. (2021). CAR T cells with dual targeting of CD19 and CD22 in pediatric and young adult patients with relapsed or refractory B cell acute lymphoblastic leukemia: a phase 1 trial. Nat. Med. *27*, 1797–1805.
51. Frimpong, K., and Spector, S.A. (2000). Cotransduction of nondividing cells using lentiviral vectors. Gene Ther. *7*, 1562–1569.
52. Spiegel, J.Y., Patel, S., Muffly, L., Hossain, N.M., Oak, J., Baird, J.H., Frank, M.J., Shiraz, P., Sahaf, B., Craig, J., et al. (2021). CAR T cells with dual targeting of CD19 and CD22 in adult patients with recurrent or refractory B cell malignancies: a phase 1 trial. Nat. Med. *27*, 1419–1431.
53. Bachiller, M., Dobaño-López, C., Rodríguez-García, A., Castellsagué, J., Gimenez-Alejandre, M., Antoñana-Vildosola, A., Martin-Antonio, B., Delgado, J., Pérez-Galán, P., Juan, M., et al. (2022). Co-Transduced CD19/BCMA Dual-Targeting CAR-T Cells for the Treatment of Non-Hodgkin Lymphoma. Blood *140*, 7386–7387.
54. Ghorashian, S., Lucchini, G., Richardson, R., Nguyen, K., Terris, C., Guvenel, A., Oporto Espuelas, M., Yeung, J., Pinner, D., Chu, J., et al. (2023). CD19/CD22 targeting with co-transduced CAR T-cells to prevent antigen negative relapse after CAR T-cell therapy of B-ALL. Blood *143*, 118–123.
55. Kokalaki, E., Ma, B., Ferrari, M., Grothier, T., Hazelton, W., Manzoor, S., Costu, E., Taylor, J., Bulek, A., Srivastava, S., et al. (2023). Dual targeting of CD19 and CD22 against B-ALL using a novel high-sensitivity aCD22 CAR. Mol. Ther. *31*, 2089–2104.
56. Bellora, F., Castriconi, R., Doni, A., Cantoni, C., Moretta, L., Mantovani, A., Moretta, A., and Bottino, C. (2012). M-CSF induces the expression of a membrane-bound form of IL-18 in a subset of human monocytes differentiating *in vitro* toward macrophages. Eur. J. Immunol. *42*, 1618–1626.
57. Jaspers, J.E., Khan, J.F., Godfrey, W.D., Lopez, A.V., Ciampricotti, M., Rudin, C.M., and Brentjens, R.J. (2023). IL-18-secreting CAR T cells targeting DLL3 are highly effective in small cell lung cancer models. J. Clin. Invest. *133*, e166028.
58. Frecha, C., Toscano, M.G., Costa, C., Saez-Lara, M.J., Cosset, F.L., Verhoeyen, E., and Martin, F. (2008). Improved lentiviral vectors for Wiskott-Aldrich syndrome gene therapy mimic endogenous expression profiles throughout haematopoiesis. Gene Ther. *15*, 930–941.
59. Maldonado-Perez, N., Tristan-Manzano, M., Justicia-Lirio, P., Martinez-Planes, E., Munoz, P., Pavlovic, K., Cortijo-Gutierrez, M., Blanco-Benitez, C., Castella, M., Juan, M., et al. (2022). Efficacy and safety of universal (TCRKO) ARI-0001 CAR-T cells for the treatment of B-cell lymphoma. Front. Immunol. *13*, 1011858.
60. Larson, R.C., Kann, M.C., Bailey, S.R., Haradhvala, N.J., Llopis, P.M., Bouffard, A.A., Scarfó, I., Leick, M.B., Grauwet, K., Berger, T.R., et al. (2022). CAR T cell killing requires the IFNgammaR pathway in solid but not liquid tumours. Nature *604*, 563–570.
61. Tristan-Manzano, M., Maldonado-Perez, N., Justicia-Lirio, P., Munoz, P., Cortijo-Gutierrez, M., Pavlovic, K., Jimenez-Moreno, R., Nogueras, S., Carmona, M.D., Sanchez-Hernandez, S., et al. (2022). Physiological lentiviral vectors for the generation of improved CAR-T cells. Mol. Ther. Oncolytics *25*, 335–349.

OMTN, Volume *35*

Supplemental information

First-in-class transactivator-free, doxycycline-inducible IL-18-engineered CAR-T cells for relapsed/refractory B cell lymphomas

Pedro Justicia-Lirio, María Tristán-Manzano, Noelia Maldonado-Pérez, Carmen Barbero-Jiménez, Marina Cortijo-Gutiérrez, Kristina Pavlovic, Francisco J. Molina-Estevez, Pilar Muñoz, Ana Hinckley-Boned, Juan R. Rodriguez-Madoz, Felipe Prosper, Carmen Griñán-Lison, Saúl A. Navarro-Marchal, Carla Panisello, Julia Muñoz-Ballester, Pedro A. González-Sierra, Concha Herrera, Juan A. Marchal, and Francisco Martín

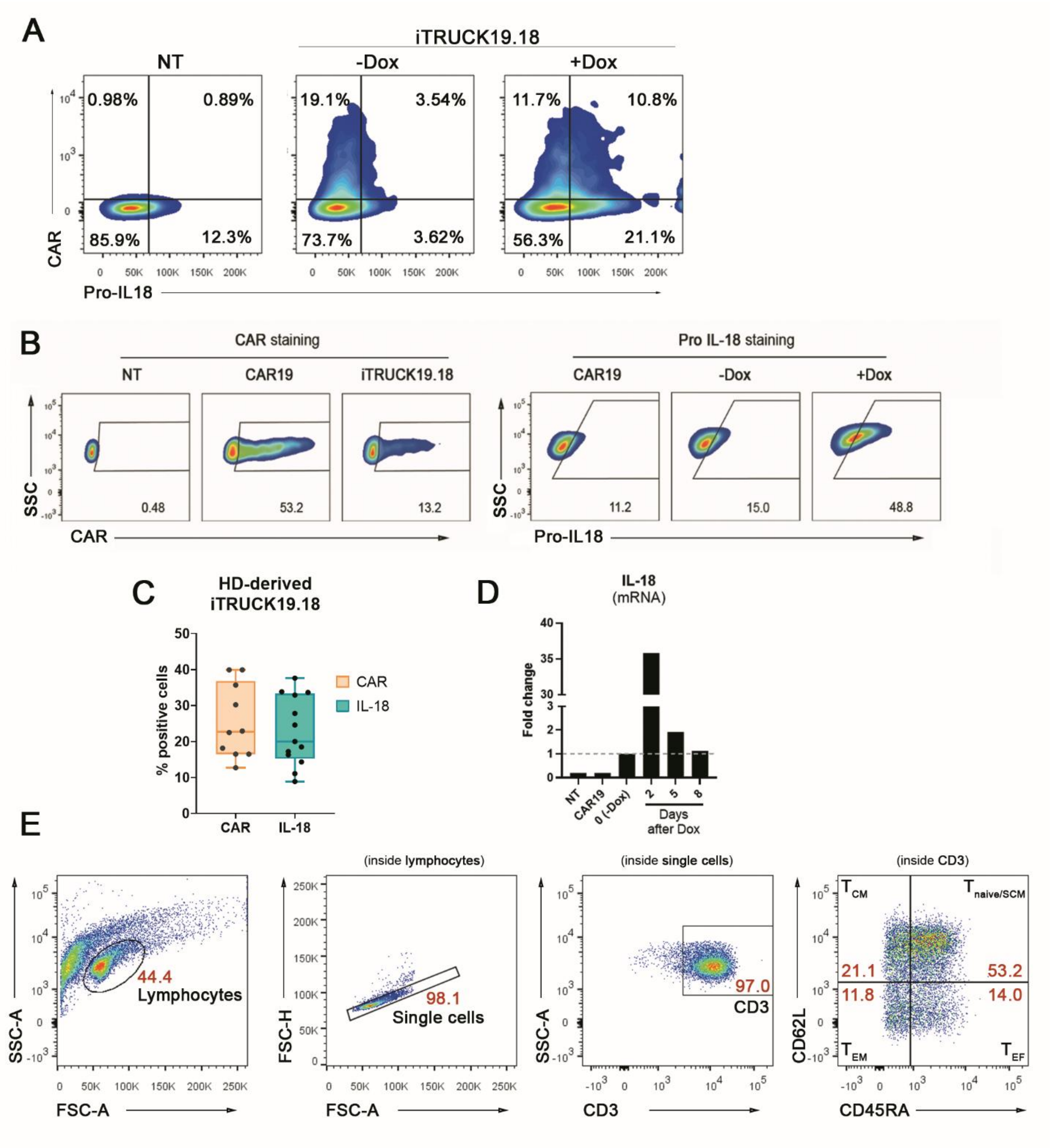

Fig. S1. Transduction efficiencies, inducibility and phenotype analysis of iTRUCK19.18 cells. (A) Representative dot-plots of co-staining of NT, iTRUCKs (-Dox) and (+Dox) 10 days after transduction. Detection was evaluated with anti-pro-IL-18 antibody (as described in M&M) and CAR-19 reagent from Miltenyi Biotech following manufacturer's instructions**.** (B) Representative dot-plots of CAR19 and iTRUCK19.18 cells showing expression of CAR (left) and pro-IL-18

(right). (C) Graph showing the percentage of CAR+ (orange bar) and IL-18 (green bar) of 10 different productions of iTRUCK19.18 cells from healthy donor, measured in the presence of Dox at day 10 post-transduction. (D) Graph showing fold change of IL-18 RNA levels in untransduced T cells, CAR19 cells and iTRUCK19.18 cells at different times upon Dox addition (2 days) and removal (days 5 and 8; days 3 and 6 without Dox). (E) Gating strategy to analyze the phenotype and subpopulations of T cells. After selecting the lymphocytes gate regarding to FSC and SSC, doubles exclusion and confirming CD3, four populations were established according to the expression of CD45RA and CD62L. $T_{naive/SCM}$: CD45RA+CD62L+; T_{CM}: CD45RA-CD62L-; T_{EM}: CD45RA-CD62L-; T_{EF}: CD45RA+CD62L-.

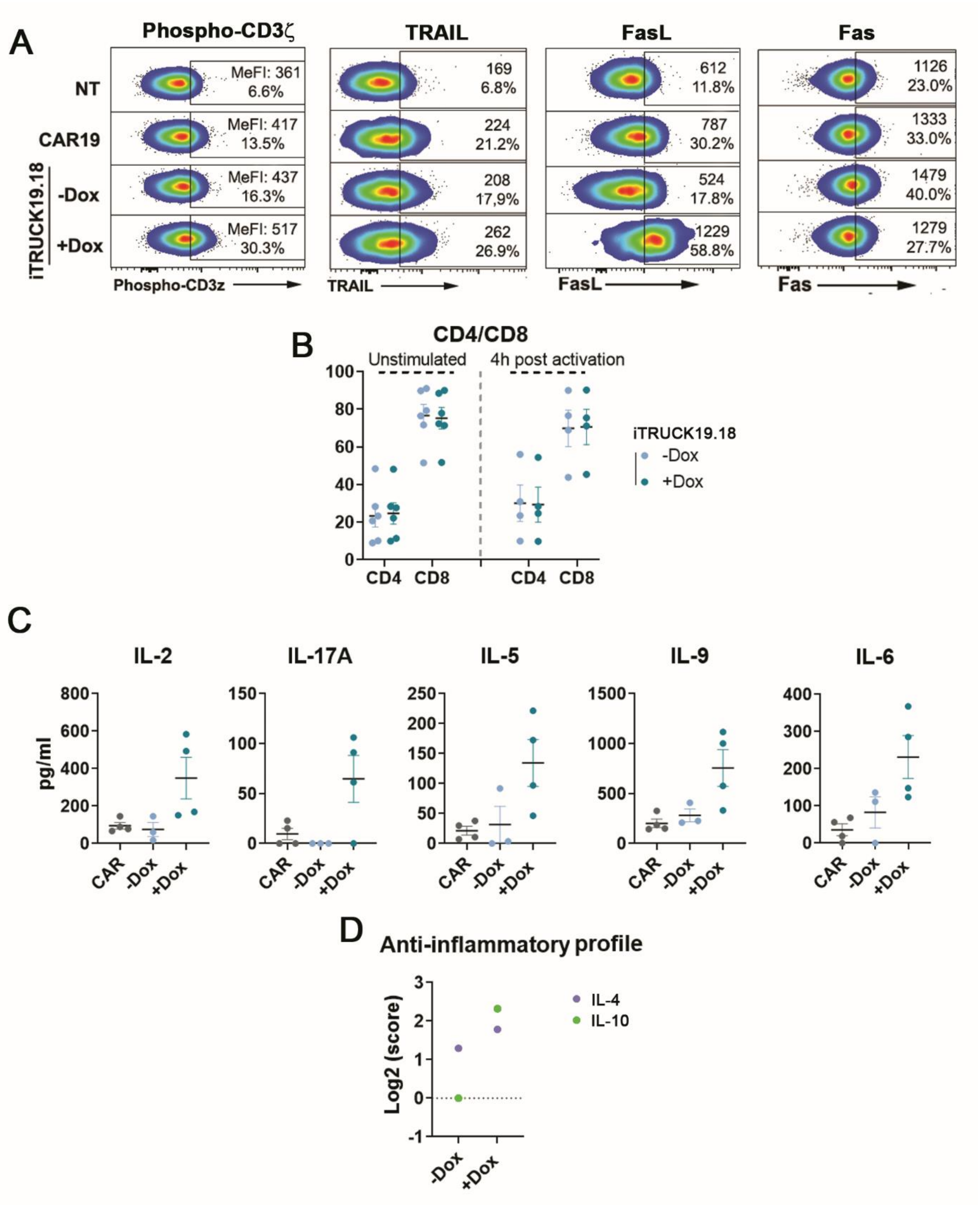

Fig. S2 Characterization of AICD markers and cytokines secretion of iTRUCK19.18 cells. (A) Representative dot-plots of phosphorylated-CD3ζ, TRAIL, FasL and Fas of untranduced T cells (NT; top Plots), CAR19 cells (second-top), iTRUCK19.18 cells -Dox (second-bottom Plots) and iTRUCK19.18 cells +Dox (+50 ng/ml Dox) (bottom plots) after manufacturing (basal

conditions). (B) Percentage of CD4/CD8 inside the iTRUCK19.18 population in the absence (-Dox) or presence of 50ng/ml of Dox (+Dox) at basal state (left; n=6) and 4h post stimulation via CD3/CD28 (right; n=4). (C) Quantification of proinflammatory cytokine secretion by iTRUCK19.18 cells (from left to right: IL-2, IL-17A, IL-5, IL-9, and IL-6) at basal state (n=4). (D) Anti-inflammatory index of iTRUCK19.18 cells (IL-4 and IL-10) at basal level (n=2) in the absence (left) and presence (right) of 50 ng/ml of Dox at basal level.

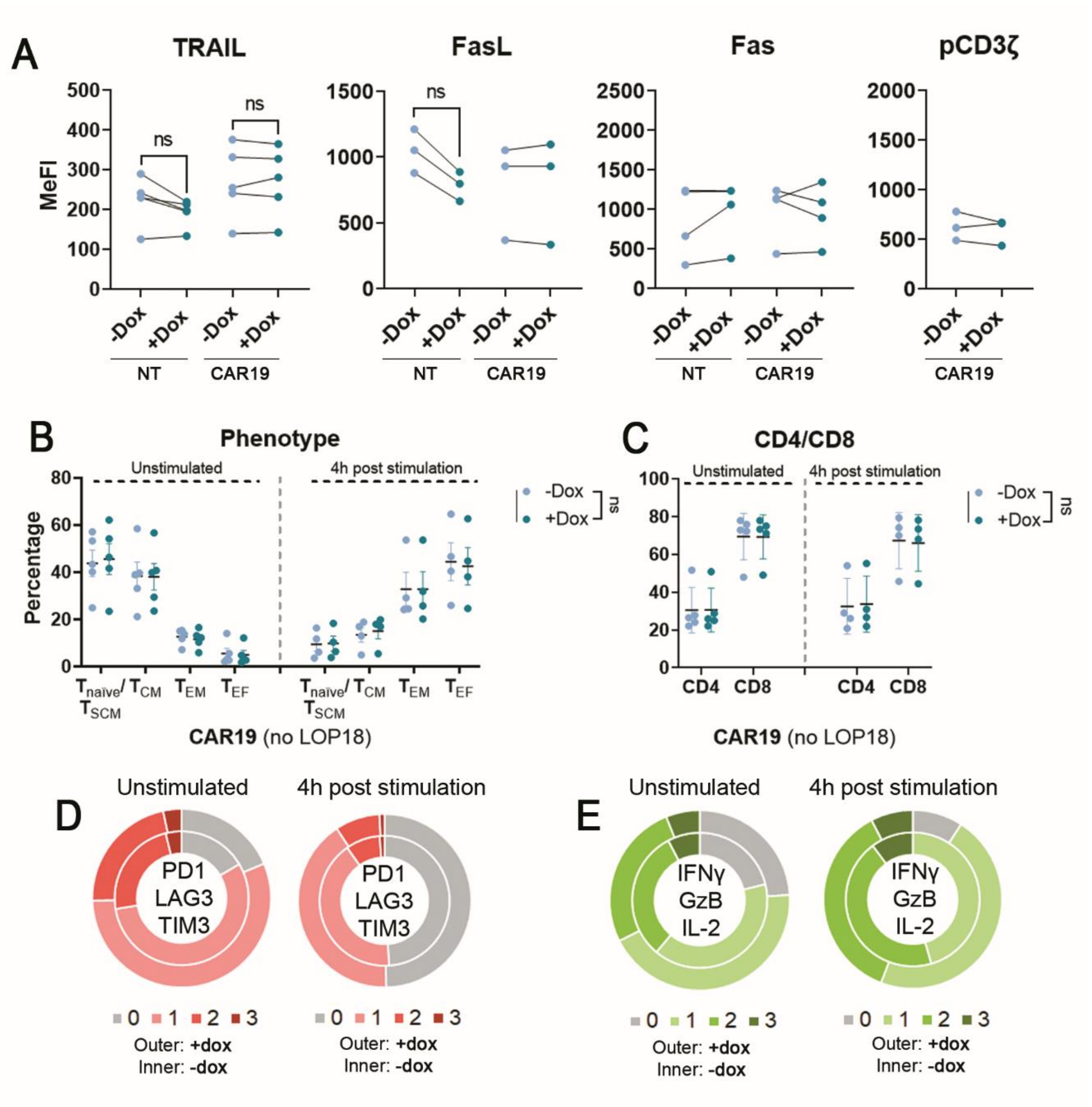

Fig. S3 Doxycycline does not alter the physiology of CAR19 cells. (A) Expression of AICD markers and phosphorylated-CD3ζ (from left to right) in NT and CAR19 cells at basal state in the presence (50 ng/ml) or absence of Dox at basal conditions after manufacturing. TRAIL (n=5), FasL (n=3), Fas (n=4) and pCD3ζ (n=3) (two-tailed paired t test). (B) Percentage of positive cells of $T_{Naïve/SCM}$, T_{CM}, T_{EM} and T_{EF} at basal state (n=5) (left) and 4h post stimulation (n=4) (right) of CAR19 cells with (50 ng/ml) and without Dox. (C) Percentage of CD4 and CD8 of CAR19 cells with (50 ng/ml) and without Dox at basal state (n=5) (left) and 4h post stimulation (n=4) (right). (D) Pie charts showing the proportion of CAR19 cells with (50 ng/ml) (outer circles) and without

(inner circles) Dox expressing 0, 1, 2, or 3 inhibitory receptors (PD1, LAG3, and TIM3) at basal state (n=4) (left) and 4h after stimulation (n=4) (right). (E) Pie charts showing the proportion of CAR19 cells with (50 ng/ml) (outer circles) and without (inner circles) Dox expressing 0, 1, 2, or 3 activation markers (IFNγ, Granzyme B, and IL-2) at basal state (n=4) (left) and 4h post stimulation (n=4) (right). Activation was performed using T cell TransAct (Miltenyi) (via CD3/CD28). ns: non-significant (two-tailed paired t test for A, one-tailed paired t test for B and C).

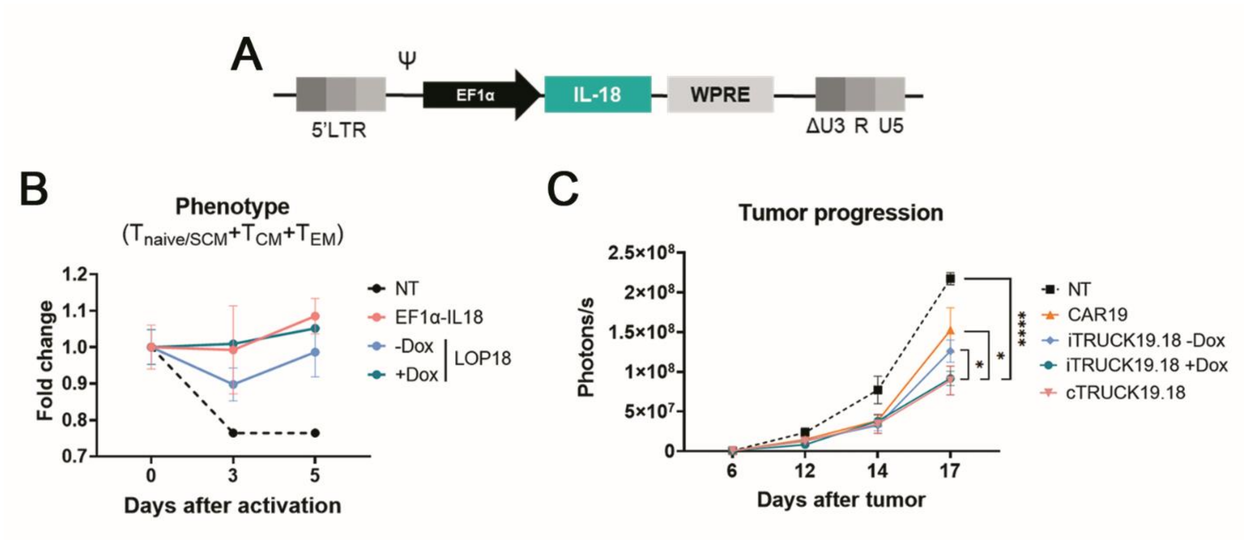

Fig S4. Phenotype and antitumor activity of CAR19 expressing IL-18 constitutively (cTRUCK19.18 cells). (A) Diagram of the lentiviral vector used for the generation of cTRUCK19.18. IL-18 is expressed under the control of EF1α promoter. (B) Phenotypic analysis of untransduced T cells (NT, black circles), cTRUCK19.18 (EF1α–IL18, orange circles), iTRUCK19.18 without Dox (LOP18 -Dox, ligh blue circles) and + 50ng/ml of Dox (LOP18 +Dox, dark blue circles) (NT: n=1; EF1α-IL-18, -Dox and +Dox: n=3). (C) Bioluminescence analysis of tumor progression *in vivo* using the Namalwa model. Once the tumor was established, mice were inoculated with $3x10^5$ CAR-T cells of CAR19 (orange triangles), cTRUCK19.18 (pink triangles), iTRUCK19.18 without Dox (light blue triangles) and in the presence of 1000ng/ml of Dox administered orally. Tumor progression was determined by bioluminescence (photons/s) of the different experimental group p<0.05, **p<0.01, ****p<0.0001 (one-tailed unpaired t test).

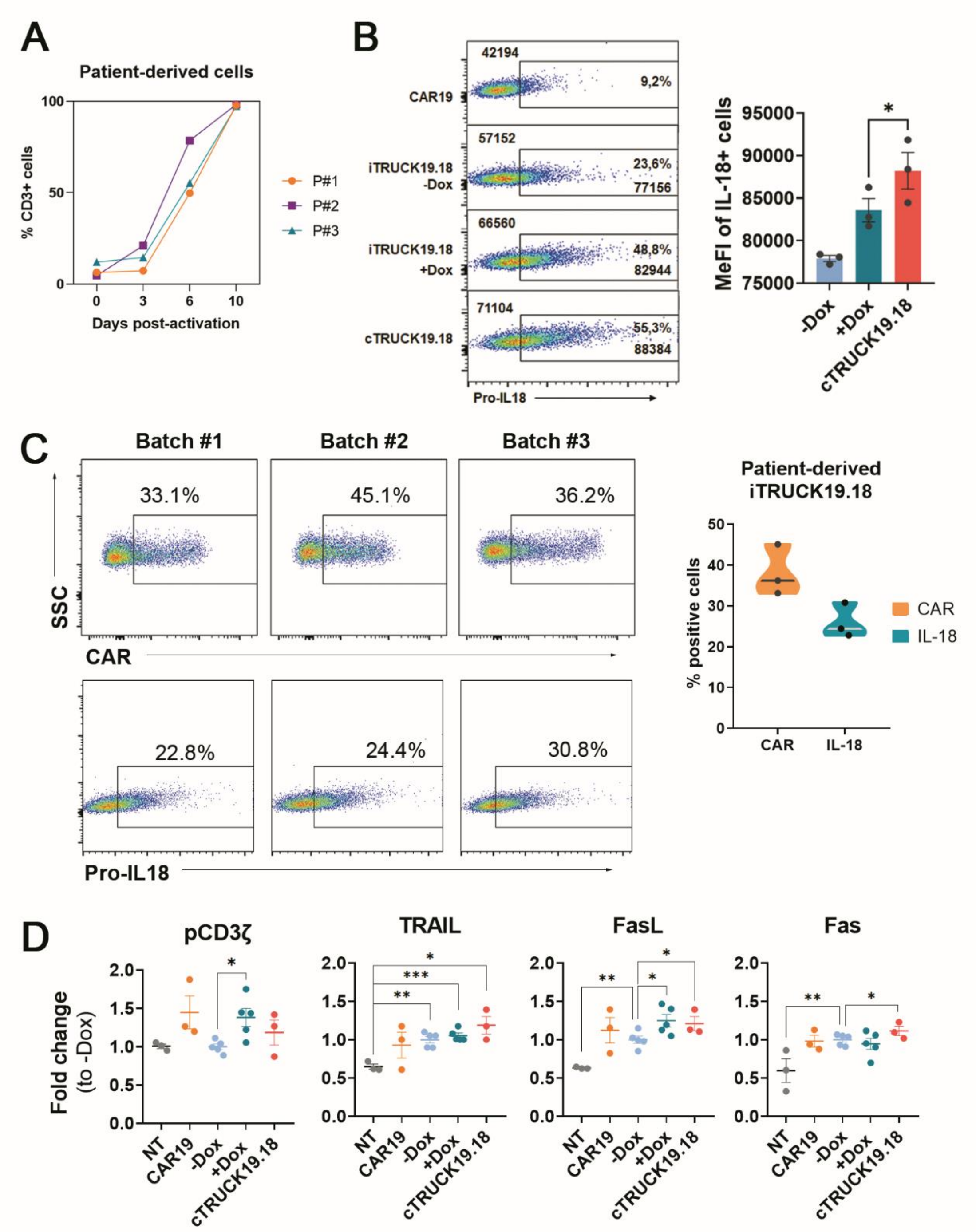

Fig. S5 Generation and characterization of patient-derived iTRUK19.18 cells. (A) Left- Enrichment of CD3+ cells from patient's #1, #2 and #3 after stimulation with TransAct during 6 days. (B) Left: representative dot-plots showing the pro-IL-18 expression levels of CAR19 (Top plot), cTRUCK19.18 (bottom plot), iTRUCK18.19 without Dox (second top plot) and iTRUCK18.19 with 50ng/ml of Dox (second bottom plot). Right. Graph showing the expression levels (as MeFI of IL-18+ cells) of cTRUCK19.18 (red bar), iTRUCK18.19 without Dox (light-

blue bar) and iTRUCK18.19 with 50ng/ml of Dox (dark-blue bar) (n=3). Paired t-test two-tails. *, $p<0.05$. (C) Left: Representative dot-plots showing CAR (top plots) and pro-IL-18 (bottom plots) expression levels of three different iTRUCK19.18 productions (batches 1,2,3) from three different patients with CD19+ malignances. Right: Graph showing variability of CAR (Orange) and pro-IL-18 (Green) expression levels of three different iTRUCK19.18 productions. All the data was collected in the presence of Dox at day 10 post-transduction (n=3). (D) Fold-change (relative to -Dox) of activation-related markers expression of patient-derived iTRUCK19.18 cells without and with (50 ng/ml). Dox treatment under basal conditions (from left to right: phospho-CD3ζ, TRAIL, FasL, and Fas). $p<0.05$, **$p<0.01$, ****$p<0.0001$ (one-tailed paired test).

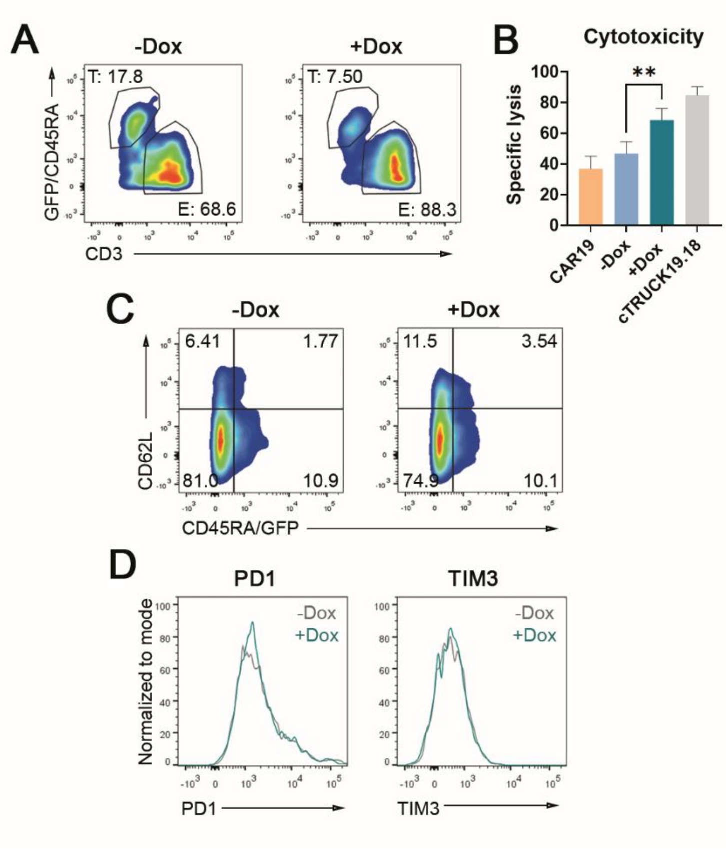

Fig. S6 Characterization of iTRUCK19.18 and cTRUCK19.18 cells. (A) Representative dot plots of iTRUCK19.18 cell lysis without (left) and with (50 ng/ml) (right) Dox from the first encounter with MIA-PaCa2 GFP-Nluc CD19+. (B) Specific lysis of CAR19, iTRUCK19.18 -Dox, iTRUCK19.18 +Dox and cTRUCK19.18 against MIA-PaCa2 GFP-Nluc at E:T ratio 1:2 (CAR19 and cTRUCK19.18: n=2; iTRUCK19.18 -Dox and +Dox: n=5. **p<0.01 (two tailed paired t test). (C) Representative dot plots of iTRUCK19.18 cell phenotype without (left) and with (50 ng/ml) (right) Dox. (D) Representative histograms of PD1 and TIM3 expression in iTRUCK19.18 cells without (left) and with (50 ng/ml) (right) Dox.

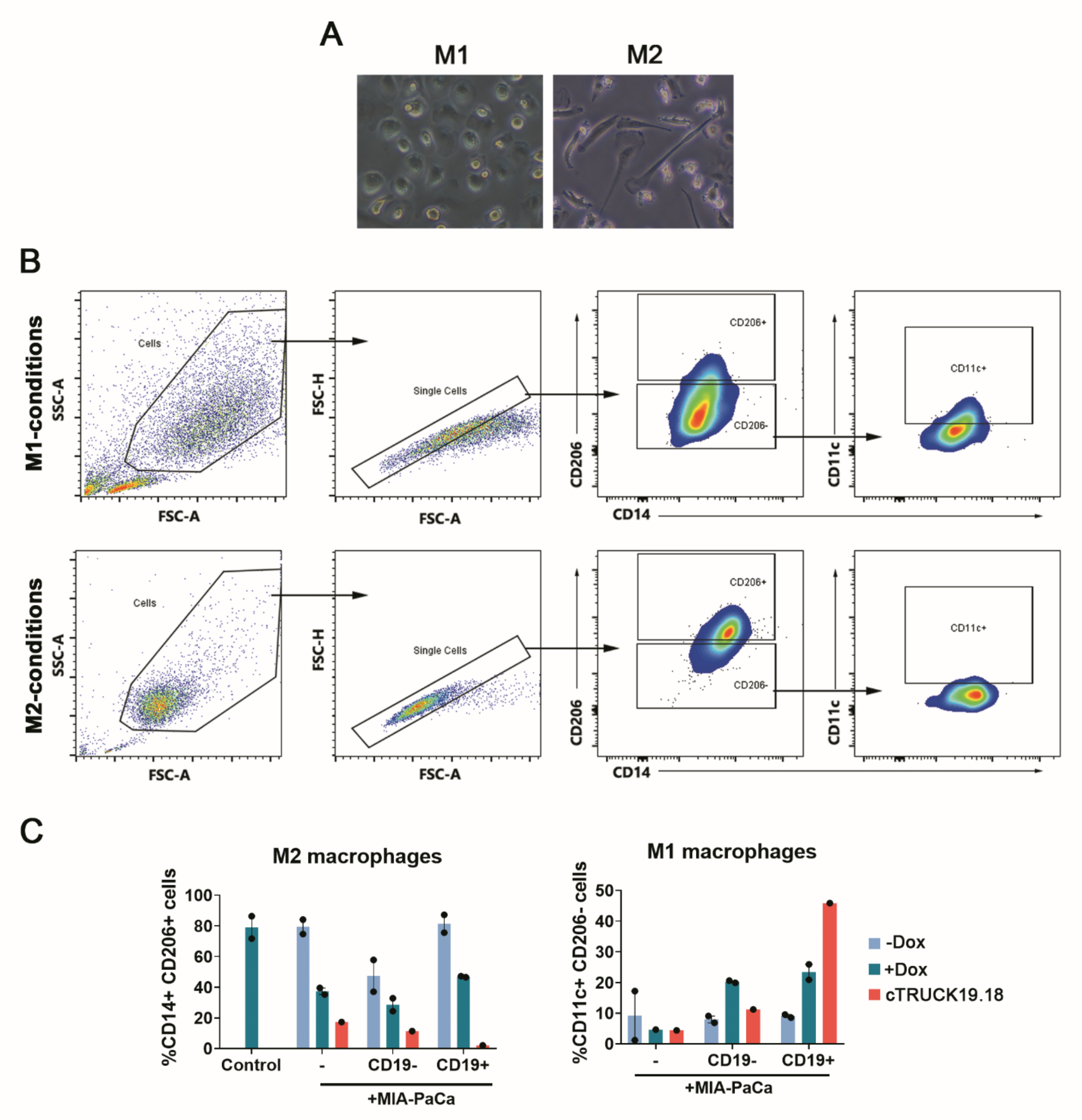

Fig. S7 Characterization of primary M1 and M2 macrophages and the effect of iTRUCK19.18 and cTRUCK19.18 on their polarization. (A) Bright-field microscopy images of human macrophages polarized to M1 (left) or M2 (right) phenotype. (B) Gating strategy for the identification of M1 and M2 macrophages by FACS according to CD14, CD206 and CD11c expression. (C) Percentage of M2 (Left; CD14+ CD206+ cells) and M1 (Right; CD11c+ cells) after the co-culture with iTRUCK19.18 (-Dox and +Dox, n=2) and cTRUCK19.18 cells (n=1) in

the absence of MIA-PACA (-) or in the presence of MIA-PaCA2-CD19 negative (CD19-) or of MIA-PaCA2-CD19+ (CD19+) during 6 days.
